# Effects of Lysine *N*-ζ-Methylation in Ultrashort Tetrabasic Lipopeptides (UTBLPs) on the Potentiation of Rifampicin, Novobiocin, and Niclosamide in Gram-Negative Bacteria

**DOI:** 10.3390/antibiotics11030335

**Published:** 2022-03-03

**Authors:** Linus Schweizer, Danyel Ramirez, Frank Schweizer

**Affiliations:** 1 Department of Laboratory Medicine and Pathobiology, University of Toronto, Toronto, ON M5S 1A8, Canada; linus.schweizer@mail.utoronto.ca; 2 Department of Chemistry, University of Manitoba, Winnipeg, MB R3T 2N2, Canada; ramiredm@myumanitoba.ca; 3 Department of Medical Microbiology and Infectious Diseases, University of Manitoba, Winnipeg, MB R3E 0J9, Canada

**Keywords:** outer membrane permeabilizer, antibiotic potentiator, antibiotic adjuvant, novobiocin, rifampicin, niclosamide, *Pseudomonas aeruginosa*, *Acinetobacter baumannii*, *Escherichia coli*

## Abstract

Outer membrane (OM) drug impermeability typically associated with a molecular weight above 600 Da and high hydrophobicity prevents accumulation of many antibiotics in Gram-negative bacteria (GNB). Previous studies have shown that ultrashort tetrabasic lipopeptides (UTBLPs) containing multiple lysine residues potentiate Gram-positive bacteria (GPB)-selective antibiotics in GNB by enhancing OM permeability. However, there is no available information on how *N*-substitution at the ζ-position of lysine in UTBLPs affects antibiotic potentiation in GNB. To study these effects, we prepared a series of branched and linear UTBLPs that differ in the degree of *N*-ζ-methylation and studied their potentiating effects with GPB-selective antibiotics including rifampicin, novobiocin, niclosamide, and chloramphenicol against wild-type and multidrug-resistant GNB isolates. Our results show that increasing *N*-ζ-methylation reduces or abolishes the potentiating effects of UTBLPs with rifampicin, novobiocin, and niclosamide against GNB. No trend was observed with chloramphenicol that is largely affected by efflux. We were unable to observe a correlation between the strength of the antibiotic potentiating effect to the increase in fluorescence in the 1-*N*-phenylnaphthylamine (NPN) OM permeability assay suggesting that other factors besides OM permeability of NPN play a role in antibiotic potentiation. In conclusion, our study has elucidated crucial structure–activity relationships for the optimization of polybasic antibiotic potentiators in GNB.

## 1. Introduction

Bacterial resistance to antibiotics is a major global health problem [1]. Currently, carbapenem-resistant *Pseudomonas aeruginosa* (CR*PA*), carbapenem-resistant *Acinetobacter baumannii* (CR*AB*), and carbapenem-resistant *Enterobacteriaceae* (CR*E*) are among the priority pathogens that pose the greatest threat to human health [2,3]. These Gram-negative bacteria (GNB) are frequently multidrug-resistant (MDR) and have become resistant to most antibiotics. Despite large investments into antibiotic discovery, no new drug class with novel modes of action against GNB has been approved in 50 years [4,5]. This failure is caused by intrinsic resistance mechanisms that prevent antibiotics from achieving the necessary intracellular concentration required to induce cell death [6]. Two major resistance mechanisms, low outer membrane (OM) permeability and efflux, control the intra-bacterial concentration of antibiotics in GNB [6,7]. In contrast to Gram-positive bacteria (GPB), GNB possess two membranes with orthogonal permeability [7]. The OM, characterized by the presence of a highly polar hydrophilic coat of lipopolysaccharides (LPS) in the outer leaflet and polar porin channels, permits the passage of only polar and low molecular weight compounds below 600 Da. In contrast, the inner membrane, devoid of LPS, typically requires non-polar and hydrophobic molecules for efficient lipid bilayer permeation [7]. Moreover, the passage of molecules through the OM is a rather slow process that when combined with elevated expression of multidrug efflux pumps, the intracellular concentration of antibiotics becomes synergistically reduced [6,7]. Recently, predictive compound accumulation rules for antibiotics have emerged for GNB like *Escherichia coli* [8]. Computational analysis of results revealed that compounds are most likely to accumulate within the cell containing one or more (primary) amino functions, and which are amphiphilic and rigid (low globularity) [8]. Unfortunately, these accumulation rules do not apply to intrinsically resistant organisms and bacteria with elevated expression of multidrug efflux pumps [8].

The OM permeability constraints of GNB prevent many hydrophobic antibiotics with a molecular weight above 600 Da from accumulating in the cell effectively. GPB-selective antibiotics such as rifampicin (RIF), novobiocin (NOV), niclosamide (NIC), and others are inactive against GNB for this reason [6,7]. However, the apparent OM impermeability of many GPB-selective antibiotics can be overcome with the use of helper molecules termed OM permeabilizers (OMPs) [9]. Ideal OMPs typically possess low antibacterial activity that is hypothesized to prevent rapid resistance development [6]. An interesting feature of OMPs is its synergistic relationship with GPB-selective antibiotics. It is theorized that OMPs destabilize the OM, resulting in an increase of the intra-bacterial concentration of antibiotics, thereby potentiating the antibacterial effect of the GPB-selective antibiotic. However, the synergistic effect of the GPB-selective antibiotic on the OMP is not understood. Over the years, several OMPs have been described, which sensitize GPB-selective antibiotics against GNB [9]. Most of the known OMPs are amphiphilic polybasic molecules that contain hydrophobic functions, and two or more basic amino or guanidino functions. Examples include peptidic OMPs derived from the polymyxin family [9,10], antimicrobial peptides [11], antimicrobial peptidomimetics [12], and amphiphilic aminoglycosides [13,14]. Recently, we have shown that ultrashort tetrabasic lipopeptides (UTBLPs) sensitize GPB-selective antibiotics against GNB [15]. For instance, it was possible to reduce the minimal inhibitory concentration (MIC) of RIF and NOV in combination with UTBLPs against CR*PA*, CR*AB*, and CR*E* to concentration levels that can be achieved in human blood, indicating that antibiotic + OMP combinations may possess therapeutic potential [15]. Moreover, preclinical and clinical investigations are in support for further validation of the antibiotic + OMP combination strategy [16,17]. For example, the human safety and pharmacokinetics of polymyxin-derived tribasic lipopeptide SPR741 were demonstrated in a Phase 1 study [18]. Perhaps the best-known OMP is PMBN, a polymyxin-based pentabasic nonapeptide devoid of the acyl chain present in polymyxins. PMBN has very weak antibacterial activity, but sensitizes many classes of antibiotics in GNB [19].

The amphiphilic character of OMPs is essential to its activity. Hydrophobicity is generally imparted by residues such as alanine, leucine, and phenylalanine among others. Incorporation of fatty acids has also been shown to enhance membrane permeabilization [20,21,22]. The acyl chain likely helps anchor the peptide into the OM by interacting with the lipid chains of the LPS [22]. Indeed, the presence of an acyl chain improves the activity of several antimicrobial peptides such as daptomycin, dalbavancin, and polymyxins [21,22]. Majority of polybasic OMPs contain primary amino and unsubstituted guanidino functions that impart polarity to the molecule. Peptidic polyamine-based OMPs can be rapidly generated by incorporation of diaminobutyric acid, ornithine, and lysine building blocks into peptides using solid or solution phase chemistry. Bivalent positively charged metal ions (Mg^2+^ or Ca^2+^) in the outer leaflet of GNB are presumably displaced by the positively charged amino groups in polyamine-based OMPs, resulting in a transient destabilization of the LPS packing of the OM, and subsequent facilitation of antibiotics into the periplasm of GNB [23].

The effect of substituted amino functions on antibacterial activity, hemolytic activity, and enzymatic degradation in polymers and peptides have been previously studied [24,25,26]. It was shown that compounds containing secondary or tertiary amines were less hemolytic than their primary amino counterparts [24,25,26]. Likewise, alkylation of the amino groups of Lys resulted in improved resistance to proteolysis [25,26]. However, it is currently unknown how the nature of the amino function (primary, secondary, tertiary, and quaternary) in peptidic OMPs affects the permeability and potentiation of GPB-selective antibiotics in GNB. To study these effects, we prepared a series of lysine-*N*-ζ-methylated ultrashort tetrabasic lipopeptides (UTBLPs) and studied their synergistic relationship with RIF, NOV, and NIC. Our data demonstrate that an increase in *N*-ζ-methylation of lysine reduces the potentiating effects of UTBLPs with GPB-selective antibiotics in GNB. These results provide critical information for the design and optimization of polyamine-based OMPs.

## 2. Results

### 2.1. Synthesis of UTBLPs ***1***–***8***

**Figure 1:**
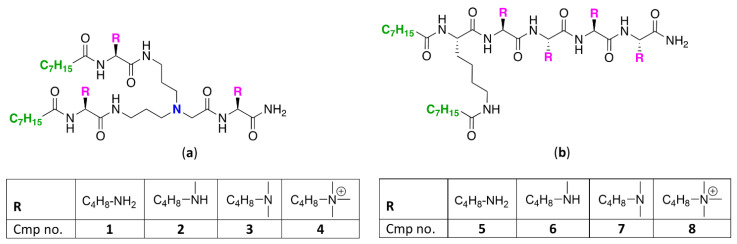
Structures of (**a**) branched and (**b**) linear UTBLPs.

To study how *N*-ζ-substitution of lysine affects the potentiation of GPB-selective antibiotics, two classes of UTBLPs were prepared (Figure 1).

Class A (compounds **1**–**4**) consists of a branched peptide backbone, while class B (compounds **5**–**8**) contains a linear peptide backbone. The branched peptide backbone was generated by incorporation of bis(3-aminopropyl)glycine into the growing peptide chain. Note that bis(3-aminopropyl) glycine also contains a tertiary amine, providing an additional protonizable group. Previous studies have shown that incorporation of bis(3-aminopropyl) glycine into UTBLP enhances the proteolytic stability of UTBLPs [15]. Lysine was selected as the parent amino acid due to the commercial availability of various *N*-ζ-methylated amino acid building blocks. A tetrabasic peptide scaffold containing two hydrophobic octanoyl residues was selected as this scaffold showed optimal potentiation of GPB-selective antibiotics and minimal cytotoxicity in previous studies [15]. The peptides were assembled using solid phase peptide synthesis (SPPS) on a Rink amide 4-methylbenzyhdrylamine (MBHA) resin following a fluorenylmethyloxycarbonyl (Fmoc) strategy [27].

### 2.2. Antibacterial Activity of UTBLPs

The standalone antibacterial activity of UTBLPs **1**–**8** was determined against a series of wild-type GNB and GPB (Table 1).

Our results demonstrate that UTBLPs **1**–**8** possess low antibacterial activity against wild-type GNB including *P. aeruginosa* PAO1, *A. baumannii* ATCC 17978, and *E. coli* ATCC 25922 (MIC ≥ 32 μg/mL). Slightly improved antibacterial activity (MIC ≥ 16 μg/mL) was seen against wild-type GPB including *Staphylococcus aureus, Enterococcus faecalis*, *and Enterococcus faecium*. Overall, the linear UTBLP **5** and **6** showed slightly improved antibacterial activity when compared to their branched counterparts **1** and **2** (Table 1). We also tested the antibacterial activity of the peptides against MDR GPB clinical isolates of *Staphylococcus epidermidis* and MDR GNB clinical isolates of *P. aeruginosa*, *A. baumannii*, *E. coli*, *Enterobacter cloacae*, and *Klebsiella pneumoniae*. Overall, these studies show that UTBLPs **1**–**8** display comparable MIC activities against MDR GNB (MIC ≥ 32 μg/mL) and GPB (MIC ≥ 8 μg/mL) when compared to wild-type organisms (Table S1).

### 2.3. Synergistic Effects of UTBLPs with RIF, NOV, NIC, and CHL against GNB

**Figure 2:**
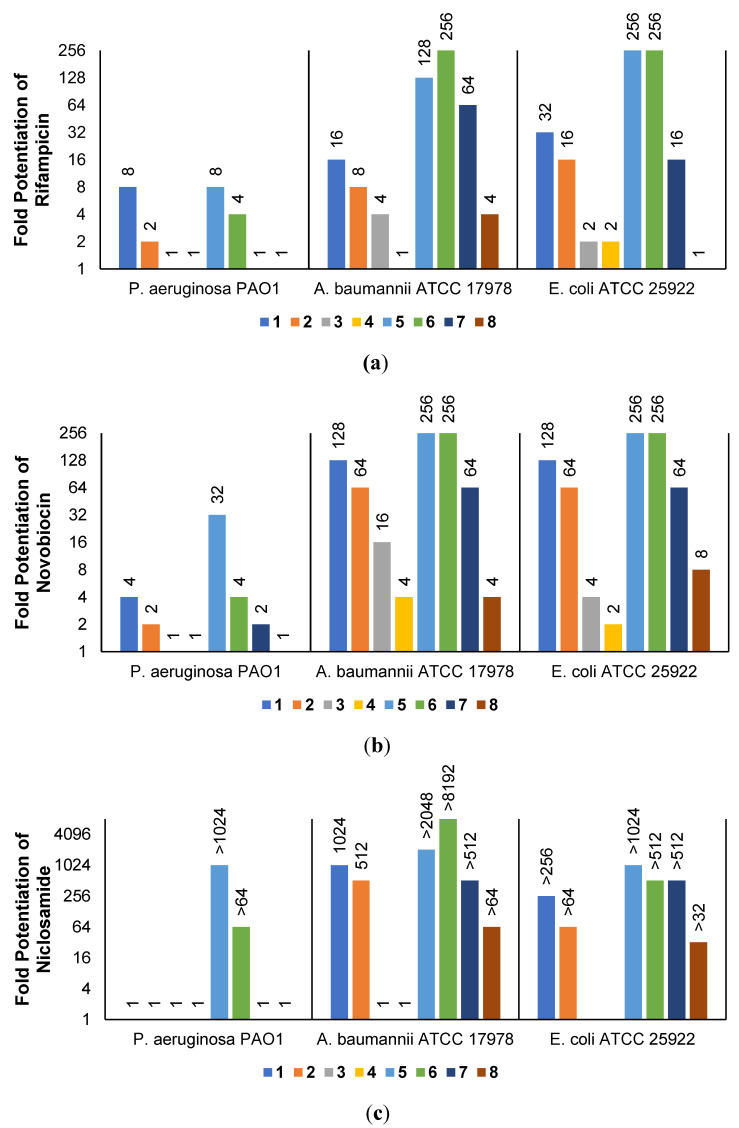

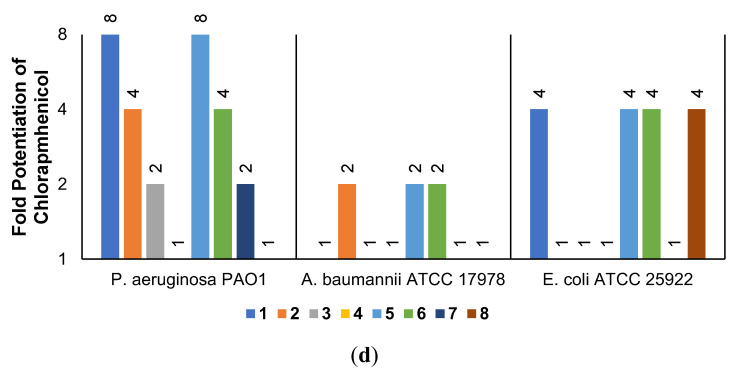
Potentiation of (**a**) RIF, (**b**) NOV, (**c**) NIC, and (**d**) CHL by 6 μM UTBLP against wild-type GNB.

**Figure 3:**
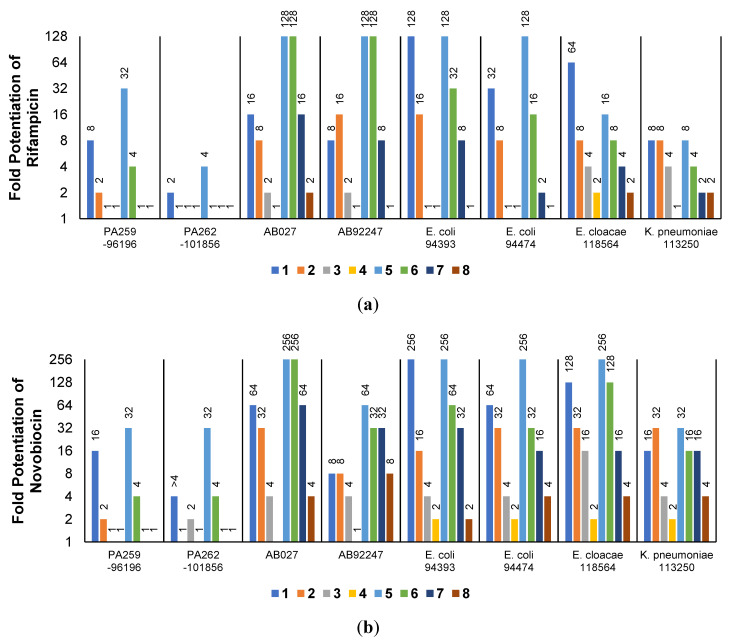
Potentiation of (**a**) RIF and (**b**) NOV by 6 μM UTBLP against a panel of MDR and carbapenem-resistant GNB isolates.

The low antibacterial activity of UTBLPs **1**–**8** prompted us to explore the potentiating effects of the compounds in combination with GPB-selective antibiotics against wild-type GNB. RIF and NOV were selected as examples of GPB-selective antibiotics as both antibiotics are inactive against GNB [15]. NIC was selected because it has been shown to synergize with polymyxins and overcome polymyxin resistance in polymyxin-resistant GNB [28]. Chloramphenicol (CHL) was selected as an antibiotic that is greatly affected by efflux [29]. The combination studies were performed by fixing the concentration of the UTBLPs to 6 μM, which is below ¼ MIC required for synergistic activity. Our results demonstrate a clear trend in the potentiating power of the various UTBLPs in combination with GPB-selective antibiotics (Figure 2). Independent of the organism and antibiotic used, the order of potentiation follows the order **1** > **2** >**3** > **4** for branched peptides and **5** > **6** > **7** > **8** for linear peptides for the antibiotics RIF and NOV (Figure 2a,b). A similar trend is seen for NIC except for *P. aeruginosa* PAO1, where no potentiation is seen for peptides **1**–**4** (Figure 2c). No clear trend can be observed with CHL except for *P. aeruginosa* PAO1 (Figure 2d). Overall, this study indicates that increasing *N*-ζ-methylation of lysine in UTBLPs decreases the potentiation of RIF, NOV, and NIC in wild-type GNB. Next, we explored whether the effects observed in wild-type GNB can be extrapolated to clinical MDR and carbapenem-resistant GNB isolates including CR*PA*, CR*AB*, and CR*E*. As shown in Figure 3a,b, we were able to confirm the same trend in MDR GNB isolates as previously observed for wild-type organisms. In general, the *N*-ζ-unmethylated UTBLPs displayed higher potentiation when compared to mono- and di-methylated compounds. The lowest potentiation was consistently observed with the tri-*N*-ζ-methylated analogs **4** and **8**. We also determined the fractional inhibitory concentration index (FICI) of compounds **1**–**8** with RIF, NOV, NIC, and CHL against wild-type and MDR GNB isolates (Tables S2 and S3) and the FICI with CHL against MDR GPB isolates (Table S4) to evaluate interactions between the two agents. FICI of ≤ 0.5, 0.5 < x ≤ 4, and > 4 were interpreted as synergy, additive, and antagonistic, respectively [30]. These results confirm that the lowest FICI (highest synergy) was consistently observed with unmethylated and mono-methylated UTBLPs when compared to the di- and tri-methylated analogs. None of the UTBLPs were able to synergize (FICI > 0.5) CHL against GPB (Table S4) confirming that UTBLPs act selectively on the OM of GNB. Moreover, the data in Table S4 indicate that UTBLPs **1**–**8** do not possess efflux pump inhibitory effects in GPB.

### 2.4. Outer Membrane Permeabilization by UTBLPs **1**–**8**

**Figure 4:**
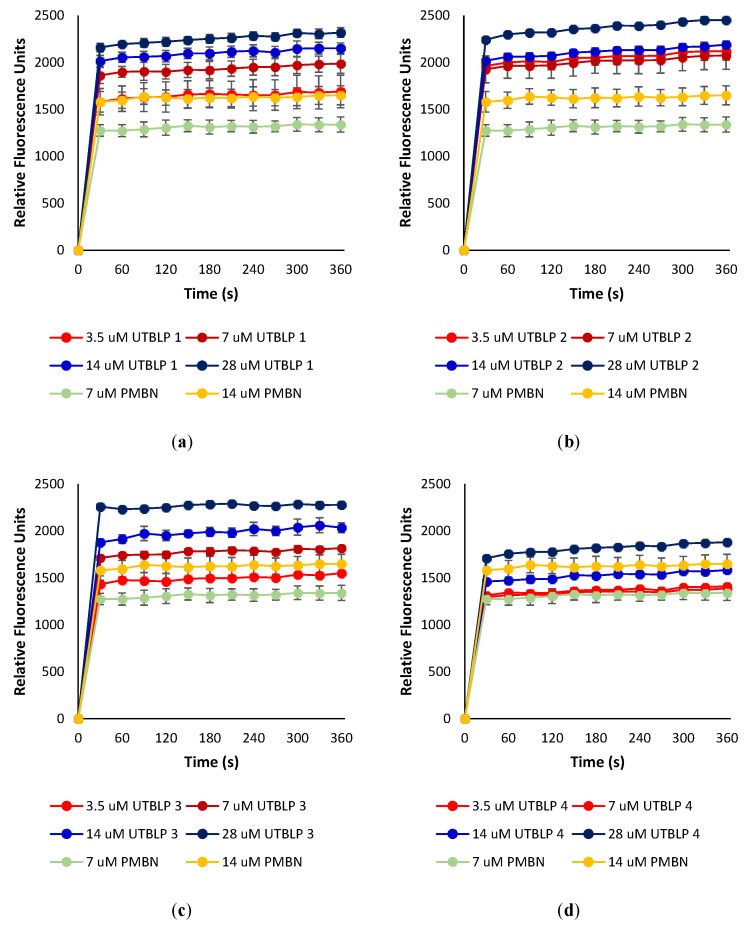
Dose-dependent increase in fluorescence of NPN in the presence of (**a**) UTBLP **1**, (**b**) UTBLP **2**, (**c**) UTBLP **3**, (**d**) UTBLP **4**, and PMBN (control) in *A. baumannii* ATCC 17978.

To determine whether UTBLPs **1**–**8** increase the intracellular concentration of RIF, NOV, and NIC by permeabilizing the OM, the ability of the peptides to increase the uptake of the nonpolar membrane-impermeable fluorescent probe 1-*N*-phenylnaphthylamine (NPN) was measured in wild-type *A. baumannii* ATCC 17978 and *E. coli* ATCC 25922. NPN uptake is normally prevented when the OM is intact [14]. Moreover, NPN fluoresces strongly and weakly in phospholipid and aqueous environments, respectively [14]. For example, exposure of *A. baumannii* ATCC 17978 to various concentrations of UTBLPs **1**–**4** or PMBN (control) resulted in a dose-dependent increase in NPN fluorescence (Figure 4). This suggests that UTBLPs **1**–**4** enhance OM permeability of the probe. Comparable dose-dependent increase in NPN fluorescence was observed with UTBLPs **1**–**4** in *E. coli* ATCC 25922 (Figure S1), as well as with UTBLPs **5**–**8** in *A. baumannii* ATCC 17978 (Figure S2) and *E. coli* ATCC 25922 (Figure S3) indicating that all UTBLPs enhance OM permeability of the probe in a dose-dependent manner. However, we were unable to correlate the antibiotic potentiation effects of UTBLPs **1**–**8** with RIF, NOV, and NIC to the increase in NPN fluorescence. For instance, the strongest antibiotic potentiators UTBLPs **1**, **2**, **5**, and **6** did not consistently produce the highest NPN fluorescence at equimolar concentrations (7 μM) (Figure S4). Moreover, the weakest potentiators **4** and **8**, which do not or only weakly potentiate GPB-selective antibiotics, displayed comparable increases in NPN fluorescence when compared to potentiators **1**, **2**, **5**, and **6**.

## 3. Discussion

The development of OMPs to potentiate GPB-selective antibiotics against clinically relevant GNB is a promising combination strategy to overcome bacterial resistance [6,9,10,16]. Majority of known OMPs are polycationic amphiphiles with two or more positive charges where the cationic character is derived from protonation of amino or guanidino functions. Polyamine-based OMPs occur as antimicrobial peptides, antimicrobial peptidomimetics, aminoglycoside-based amphiphiles, cationic lipids, and detergents [6,9,10,12,13,14,15,16]. Polyamine-based OMPs are believed to destabilize the OM by displacing bivalent cations (Mg^2+^ or Ca^2+^), which are stabilizing counterions for the phosphate groups of lipid A and the phosphorylated core sugars that prevent repulsion between and among individual LPS molecules. This leads to a localized disruption of LPS in the OM allowing non-porin mediated passage of antibiotics into the periplasm [19,23,31,32]. Prior to this study, there was no information available on how *N*-alkylation of polyamine-based OMPs affects the potentiation of GPB-selective antibiotics. We studied this effect for the first time in a systematic fashion by preparing a series of UTBLPs via incorporation of various *N*-ζ-methylated lysine analogs into branched and linear peptide backbone scaffolds. Our results demonstrate that an increase in *N*-ζ-methylation in UTBLPs reduces the potentiation of RIF, NOV, and NIC in clinically relevant wild-type and MDR GNB. Hydrophobicity and a molecular weight above 600 Da, such as in the antibiotics RIF and NOV, reduce or prevent porin-mediated permeation through the OM. In contrast, NIC is a hydrophobic GPB-selective antibiotic with a molecular weight below 600 Da [28]. Previous studies have shown that OMPs, including PMBN and polymyxin-based antibiotics, strongly potentiate NIC against GNB indicating that the low activity of NIC against GNB is caused by low OM permeability [28]. Interestingly, none of the branched UTBLPs **1**–**4** potentiate NIC in *P. aeruginosa* PAO1. This suggests different requirements for UTBLPs to potentiate NIC against *P. aeruginosa* PAO1 when compared to other wild-type GNB. In contrast to RIF, NOV, and NIC, no potentiation trend can be observed with UTBLPs **1**–**8** in combination with CHL against *A. baumannii* ATCC 17978 and *E. coli* ATCC 25922. This likely reflects that OM permeability of CHL is less important for these organisms when compared to *P. aeruginosa* PAO1. The fact that CHL is potentiated in *P. aeruginosa* PAO1 by UTBLPs in the order **1** > **2** > **3** > **4** and **5** > **6** > **7** > **8** reflects the greatly reduced OM permeability of *P. aeruginosa* when compared to other GNB [24,25]. Moreover, we were unable to correlate the antibiotic potentiation effects of equimolar UTBLPs **1**–**8** with RIF, NOV, and NIC to an increase in NPN fluorescence in the OM permeability assay. This indicates that other factors besides OM permeability control the potentiation effects of RIF, NOV, and NIC in GNB. Our study shows that the nature of the peptide backbone can influence the potentiation effect of RIF, NOV, and NIC. For instance, we typically observed a two- to four-fold increase in potentiation of antibiotics in the linear UTBLPs **5**–**8** when compared to the branched UTBLPs **1–4**. Whether the reduced potentiating effects of the branched UTBLPs **1** and **2** reflects different peptide backbone topology or reflects a reduced number of primary or secondary amino groups in UTBLPs **1** and **2** when compared to **5** and **6** needs to be further explored. In summary, our results indicate that the most effective antibiotic potentiator molecules to potentiate RIF, NOV, and NIC against GNB are UTBLPs with three or more primary amino functions.

## 4. Materials and Methods

### 4.1. Materials

The Rink amide MBHA resin, Fmoc-L-Lys(Me)_3_-OH, and Fmoc-L-Lys(Fmoc)-OH were purchased from Sigma-Aldrich (St Louis, MO, USA). Fmoc-L-Lys(Boc)-OH was purchased from AK Scientific (Union City, CA, USA), Fmoc-L-Lys(Me,Boc)-OH from Biosynth Carbosynth (Newbury, UK), Fmoc-L-Lys(Me)_2_-OH from Bachem (Bubendorf, Switzerland), and *N*,*N*-bis(N′-Fmoc-3-aminopropyl)glycine potassium hemisulfate from Chem-Impex (Wood Dale, IL, USA). All other reagents and solvents were obtained from commercial sources and used without further purification.

### 4.2. Preparation of UTBLPs 1-8

All UTBLPs were prepared by Fmoc SPPS [27] and as previously described [15,33]. The N-terminus of the amino acids was protected with Fmoc. The-*N*-ζ-amino function in unmethylated or *N*-ζ-monomethylated Lys were protected with *tert*-butyloxycarbonyl (Boc), while no side chain protecting group was necessary for *N*-ζ-dimethylated and *N*-ζ-trimethylated Lys building blocks. The Rink amide MBHA resin was loaded on the peptide synthesis vessel and was subjected to 20% piperidine to remove Fmoc. The first protected amino acid (3 mol. eq.) was mixed with *O-*(benzotriazol-1-yl)-*N*,*N*,*N’*,*N’*-tetramethyluronium tetrafluoroborate (3 mol. eq.), and *N*-methylmorpholine (8 mol. eq.) in DMF for 5 min. Once the Fmoc-amino acid was activated, the solution was transferred to the resin and gently agitated with nitrogen gas for 45 min. The Fmoc removal and coupling steps were repeated for the addition of the succeeding amino acids and fatty acids. The chloranil test (2% chloranil in DMF) was performed on a small amount of resin to determine whether the reactions reached completion. Deprotection of sidechain protecting groups and peptide cleavage was achieved using 95% trifluoroacetic acid (TFA) affording solid compounds as TFA salts.

The crude was purified via reverse-phase flash chromatography using a solvent system of 55% methanol spiked with 0.1% TFA. Chemical characterization and purity of the compounds were assessed with nuclear magnetic resonance (NMR) spectroscopy on a Bruker AMX-500 (Germany), and matrix-assisted laser desorption ionization mass spectrometry on a Bruker Ultraflextreme (Germany) or by electrospray ionization mass spectrometry on a Bruker Compact (Germany). The NMR spectra are provided in the Supplementary Material. 

#### 4.2.1. Chemical Characterization of UTBLP 1

^1^H NMR (500 MHz, MeOD) δ 4.38–4.29 (m, 1H, Lys_1_-α), 4.23–4.12 (m, 2H, Lys_2,3_-α), 4.10–3.98 (m, 2H, Linker-1), 3.43–3.31 (m, 2H, Linker-2,4), 3.29–3.15 (m, 6H, Linker-2,4), 3.02–2.85 (m, 6H, Lys_1,2,3_-ε), 2.34–2.18 (m, 4H, Aliphatic-a), 1.99–1.88 (m, 4H, Linker-3), 1.88–1.76 (m, 3H, Lys_1,2,3_-β), 1.76–1.63 (m, 9H, Lys_1,2,3_-β + Lys_1,2,3_-δ), 1.63–1.56 (m, 4H, Aliphatic-b), 1.54–1.38 (m, 6H, Lys_1,2,3_-γ), 1.37–1.21 (m, 16H, Aliphatic-c), 0.94–0.83 (m, 6H, Aliphatic-d).

^13^C NMR (126 MHz, MeOD) δ 175.25, 174.67, 173.89, 164.82 (carbonyl), 53.75 (Lys_2,3_-α), 53.53 (Linker-1), 53.45 (Lys_1_-α), 53.05 (Linker-2,4), 39.01 (Lys_2,3_-ε), 38.99 (Lys_1_-ε), 35.53 (Linker-2,4), 35.48 (Aliphatic-a), 31.47 (Aliphatic-c), 31.02, 30.78 (Lys_1,2,3_-β + Lys_1,2,3_-δ), 28.95, 28.71 (Aliphatic-c), 26.75, 26.70 (Lys_1,2,3_-β + Lys_1,2,3_-δ), 25.47 (Aliphatic-b), 23.95 (Linker-3), 22.59 (Lys_2,3_-γ), 22.39 (Lys_1_-γ), 22.24 (Aliphatic-c), 12.98 (Aliphatic-d).

MS (+TOF) *m*/*z*: calculated for C_42_H_84_N_10_O_6_ [M+H]^+^: 825.665, found: 825.766; [M+Na]^+^: 847.647, found: 847.762.

#### 4.2.2. Chemical Characterization of UTBLP 2

^1^H NMR (500 MHz, MeOD) δ 4.35–4.31 (m, 1H, Lys_1_-α), 4.22–4.15 (m, 2H, Lys_2,3_-α), 4.08–3.99 (m, 2H, Linker-1), 3.48–3.31 (m, 2H, Linker-2 + Linker-4), 3.27–3.09 (m, 6H, Linker-2 + Linker-4), 3.02–2.95 (m, 6H, Lys_1,2,3_-ε), 2.70–2.65 (m, 9H, methyl), 2.28–2.21 (m, 4H, Aliphatic-a), 1.91 (m, 4H, Linker-3), 1.85–1.64 (m, 12H, Lys_1,2,3_-β + Lys_1,2,3_-δ), 1.62–1.55 (m, 4H, Aliphatic-b), 1.53–1.39 (m, 6H, Lys_1,2,3_-γ), 1.35–1.27 (m, 16H, Aliphatic-c), 0.92–0.86 (m, 6H, Aliphatic-d).

^13^C NMR (126 MHz, MeOD) δ 175.24, 174.58, 173.84, 164.80 (carbonyl), 53.68 (Lys_2,3_-α), 53.54 (Linker-1), 53.39 (Lys_1_-α), 53.06 (Linker-2,4), 48.64 (Lys_2,3_-ε), 48.60 (Lys_1_-ε), 35.53 (Linker-2,4), 35.39 (Aliphatic-a), 32.12 (methyl), 31.47 (Aliphatic-c), 31.03, 30.77 (Lys_1,2,3_-β + Lys_1,2,3_-δ), 28.96, 28.72 (Aliphatic-c), 25.48 (Aliphatic-b), 25.32, 25.26 (Lys_1,2,3_-β + Lys_1,2,3_-δ), 23.96 (Linker-3), 22.59 (Lys_2,3_-γ), 22.39 (Lys_1_-γ), 22.24 (Aliphatic-c), 12.98 (Aliphatic-d).

MS (+TOF) *m*/*z*: calculated for C_45_H_90_N_10_O_6_ [M+H]^+^: 867.712, found: 867.800; [M+Na]^+^: 889.694, found: 889.797; [M+K]^+^: 905.803, found: 905.793.

#### 4.2.3. Chemical Characterization of UTBLP 3

^1^H NMR (500 MHz, MeOD) δ 4.36–4.31 (m, 1H, Lys_1_-α), 4.23–4.16 (m, 2H, Lys_2,3_-α), 4.12–3.94 (m, 2H, Linker-1), 3.38–3.30 (m, 2H, Linker-2 + Linker-4), 3.29–3.16 (m, 6H, Linker-2 + Linker-4), 3.15–3.08 (m, 6H, Lys_1,2,3_-ε), 2.88–2.85 (m, 18H, methyl), 2.31–2.22 (m, 4H, Aliphatic-a), 1.96–1.88 (m, 4H, Linker-3), 1.87–1.65 (m, 12H, Lys_1,2,3_-β + Lys_1,2,3_-δ), 1.63–1.56 (m, 4H, Aliphatic-b), 1.52–1.38 (m, 6H, Lys_1,2,3_-γ), 1.35–1.25 (m, 16H, Aliphatic-c), 0.96–0.84 (m, 6H, Aliphatic-d).

^13^C NMR (126 MHz, MeOD) δ 175.22, 174.58, 173.79, 164.80 (carbonyl), 57.24 (Lys_2,3_-ε), 57.20 (Lys_1_-ε), 53.61 (Lys_2,3_-α), 53.54 (Linker-1), 53.37 (Lys_1_-α), 53.08 (Linker-2,4), 41.97 (methyl), 35.54 (Linker-2,4), 35.40 (Aliphatic-a), 31.47 (Aliphatic-c), 31.01, 30.76 (Lys_1,2,3_-β + Lys_1,2,3_-δ), 28.97, 28.72 (Aliphatic-c), 25.48 (Aliphatic-b), 23.94 (Linker-3), 23.72, 23.63 (Lys_1,2,3_-β + Lys_1,2,3_-δ), 22.53 (Lys_2,3_-γ), 22.35 (Lys_1_-γ), 22.24 (Aliphatic-c), 12.98 (Aliphatic-d).

MS (+TOF) *m*/*z*: calculated for C_48_H_96_N_10_O_6_ [M+H]^+^: 909.759, found: 909.849; [M+Na]^+^: 931.503, found: 931.837; [M+K]^+^: 947.850, found: 947.809.

#### 4.2.4. Chemical Characterization of UTBLP 4

^1^H NMR (500 MHz, MeOD) δ 4.36–4.32 (m, 1H, Lys_1_-α), 4.23–4.16 (m, 2H, Lys_2,3_-α), 4.09–3.99 (m, 2H, Linker-1), 3.46–3.32 (m, 6H, Lys_1,2,3_-ε), 3.32–3.31 (m, 1H, Linker-2 + Linker-4), 3.29–3.19 (m, 7H, Linker-2 + Linker-4), 3.15–3.08 (m, 27H, methyl), 2.30–2.21 (m, 4H, Aliphatic-a), 1.96–1.90 (m, 4H, Linker-3), 1.89–1.67 (m, 12H, Lys_1,2,3_-β + Lys_1,2,3_-δ), 1.64–1.56 (m, 4H, Aliphatic-b), 1.51–1.37 (m, 6H, Lys_1,2,3_-γ), 1.35–1.25 (m, 16H, Aliphatic-c), 0.93–0.84 (m, 6H, Aliphatic-d).

^13^C NMR (126 MHz, MeOD) δ 175.21, 174.48, 173.72, 164.80 (carbonyl), 66.06 (Lys_2,3_-ε), 66.01 (Lys_1_-ε), 53.58 (Lys_2,3_-α), 53.51 (Linker-1), 53.39 (Lys_1_-α), 53.10 (Linker-2,4), 52.13 (methyl), 52.11 (methyl), 52.07 (methyl), 35.56 (Linker-2,4), 35.42 (Aliphatic-a), 31.48 (Aliphatic-c), 31.07, 30.79 (Lys_1,2,3_-β + Lys_1,2,3_-δ), 28.98, 28.73 (Aliphatic-c), 25.50 (Aliphatic-b), 23.96 (Linker-3), 22.44, 22.25 (Lys_1,2,3_-β + Lys_1,2,3_-δ), 22.18 (Lys_2,3_-γ), 22.13 (Lys_1_-γ), 22.07 (Aliphatic-c), 12.98 (Aliphatic-d).

MS (+TOF) *m*/*z*: calculated for C_51_H_105_N_10_O_6_^3+^ [M+TFA]^2+^: 533.403, found: 533.398.

#### 4.2.5. Chemical Characterization of UTBLP 5

^1^H NMR (500 MHz, MeOD) δ 4.34–4.24 (m, 4H, Lys_1,2,3,4_-α), 4.17–4.12 (m, 1H, Lys_5_-α), 3.17–3.12 (m, 2H, Lys_5_-ε), 2.98–2.89 (m, 8H, Lys_1,2,3,4_-ε), 2.29–2.21 (m, 2H, Aliphatic_1_-a), 2.16 (m, 2H, Aliphatic_2_-a), 1.90–1.81 (m, 4H, Lys_2,3,4,5_-β), 1.79–1.72 (m, 4H, Lys_2,3,4,5_-β), 1.71–1.62 (m, 10H, Lys_1_-β + Lys_1,2,3,4_-δ), 1.62–1.55 (m, 4H, Aliphatic_1,2_-b), 1.54–1.35 (m, 12H, Lys_1,2,3,4,5_-γ + Lys_5_-δ), 1.34–1.25 (m, 16H, Aliphatic_1,2_-c), 0.93–0.85 (m, 6H, Aliphatic_1,2_-d).

^13^C NMR (126 MHz, MeOD) δ 175.48, 175.12, 174.92, 173.84, 172.96, 172.91, 172.58 (carbonyl), 54.22 (Lys_5_-α), 53.47, 53.42, 53.37, 52.78 (Lys_1,2,3,4_-α), 39.17, 39.12, 39.07, 39.02 (Lys_1,2,3,4_-ε), 38.51 (Lys_5_-ε), 35.77 (Aliphatic_2_-a), 35.31 (Aliphatic_1_-a), 31.48, 31.45 (Aliphatic_1,2_-c), 31.07, 30.70, 30.68, 30.65, 30.42 (Lys_1,2,3,4,5_-β), 28.92, 28.87, 28.71, 28.69, 28.65 (Lys_5_-δ + 4 Aliphatic_1,2_-c), 26.59, 26.54, 26.51, 26.43 (Lys_1,2,3,4_-δ), 25.69, 25.49 (Aliphatic_1,2_-b), 22.87, 22.54, 22.41, 22.32, 22.30, 22.23, 22.22 (Lys_1,2,3,4,5_-γ + 2 Aliphatic-c), 12.98, 12.97 (Aliphatic_1,2_-d).

MS (+TOF) *m*/*z*: calculated for C_46_H_91_N_11_O_7_ [M+H]^+^: 910.718, found: 910.718; [M+Na]^+^: 933.700, found: 932.700; [M+2H]^2+^: 455.862, found: 455.867.

#### 4.2.6. Chemical Characterization of UTBLP 6

^1^H NMR (500 MHz, MeOD) δ 4.35–4.20 (m, 4H, Lys_1,2,3,4_-α), 4.18–4.12 (m, 1H, Lys_5_-α), 3.18–3.11 (m, 2H, Lys_5_-ε), 3.07–2.88 (m, 8H, Lys_1,2,3,4_-ε), 2.81–2.55 (m, 12H, methyl), 2.30–2.20 (m, 2H, Aliphatic_1_-a), 2.19–2.13 (m, 2H, Aliphatic_2_-a), 1.95–1.82 (m, 4H, Lys_2,3,4,5_-β), 1.82–1.73 (m, 4H, Lys_2,3,4,5_-β), 1.73–1.64 (m, 10H, Lys_1_-β + Lys_1,2,3,4_-δ), 1.62–1.55 (m, 4H, Aliphatic_1,2_-b), 1.55–1.35 (m, 12H, Lys_1,2,3,4,5_-γ + Lys_5_-δ), 1.35–1.22 (m, 16H, Aliphatic_1,2_-c), 0.99–0.75 (m, 6H, Aliphatic_1,2_-d).

^13^C NMR (126 MHz, MeOD) δ 175.38, 175.05, 174.89, 173.73, 172.83, 172.77, 172.51 (carbonyl), 54.12 (Lys_5_-α), 53.25, 53.23, 53.20, 52.77 (Lys_1,2,3,4_-α), 48.69, 48.66, 48.64, 48.59 (Lys_1,2,3,4_-ε), 38.51 (Lys_5_-ε), 35.78 (Aliphatic_2_-a), 35.34 (Aliphatic_1_-a), 32.21, 32.19, 32.17, 32.15 (methyl), 31.48, 31.46 (Aliphatic_1,2_-c), 31.09, 30.75, 30.70, 30.48, 30.31, 28.93, 28.88, 28.73, 28.70 (Lys_1,2,3,4,5_-β + 4 Aliphatic_1,2_-c), 25.70, 25.57, 25.53, 25.49, 25.16, 25.12, 25.09 (Lys_1,2,3,4,5_-δ + 2 Aliphatic_1,2_-b), 22.89, 22.49, 22.41, 22.37, 22.29, 22.24, 22.23 (Lys_1,2,3,4,5_-γ + 2 Aliphatic_1,2_-c), 12.98, 12.97 (Aliphatic_1,2_-d). 

MS (+TOF) *m*/*z*: calculated for C_50_H_99_N_11_O_7_ [M+H]^+^: 966.780, found: 966.781; [M+2H]^2+^: 483.894, found: 483.897.

#### 4.2.7. Chemical Characterization of UTBLP 7

^1^H NMR (500 MHz, MeOD) δ 4.33–4.25 (m, 4H, Lys_1,2,3,4_-α), 4.19–4.13 (m, 1H, Lys_5_-α), 3.16–3.09 (m, 10H, Lys_1,2,3,4,5_-ε), 2.88–2.86 (m, 24H, methyl), 2.30–2.22 (m, 2H, Aliphatic_1_-a), 2.19–2.14 (m, 2H, Aliphatic_2_-a), 1.91–1.84 (m, 4H, Lys_2,3,4,5_-β), 1.82–1.76 (m, 4H, Lys_2,3,4,5_-β), 1.75–1.64 (m, 10H, Lys_1_-β + Lys_1,2,3,4_-δ), 1.62–1.56 (m, 4H, Aliphatic_1,2_-b), 1.53–1.39 (m, 12H, Lys_1,2,3,4,5_-γ + Lys_5_-δ), 1.34–1.27 (m, 16H, Aliphatic_1,2_-c), 0.91–0.87 (m, 6H, Aliphatic_1,2_-d).

^13^C NMR (126 MHz, MeOD) δ 175.31, 174.97, 174.87, 173.66, 172.76, 172.68, 172.44 (carbonyl), 57.37, 57.35, 57.27, 57.17 (Lys_1,2,3,4_-ε), 57.16 (Lys_5_-α), 54.04, 53.14, 53.11, 52.73 (Lys_1,2,3,4_-α), 42.15, 42.12, 42.03, 41.99, 41.90, 41.88, 41.84, 41.81, 38.52 (Lys_5_-ε), 35.79 (Aliphatic_2_-a), 35.38 (Aliphatic_1_-a), 31.48, 31.46, 31.12, 30.85, 30.81, 30.76, 30.56, 28.94, 28.88, 28.74, 28.70 (Lys_1,2,3,4,5_-β + 4 Aliphatic_1,2_-c), 25.70, 25.55 (Lys_1,2,3,4,5_-δ + 2 Aliphatic_1,2_-b), 23.67, 23.63, 23.59, 23.57, 23.53, 22.92, 22.40, 22.36, 22.33, 22.25, 22.23, 22.21 (Lys_1,2,3,4,5_-γ + 2 Aliphatic_1,2_-c), 12.99, 12.97 (Aliphatic_1,2_-d). 

MS (+TOF) *m*/*z*: calculated for C_54_H_107_N_11_O_7_ [M+H]^+^: 1022.843, found: 1022.828; [M+Na]^+^: 1044.825, found: 1044.810; [M+2H]^2+^: 511.925, found: 511.919; [M+2Na]^2+^: 533.908, found: 533.397.

#### 4.2.8. Chemical characterization of UTBLP 8

^1^H NMR (500 MHz, MeOD) δ 4.38–4.28 (m, 4H, Lys_1,2,3,4_-α), 4.21–4.15 (m, 1H, Lys_5_-α), 3.38–3.30 (m, 8H, Lys_1,2,3,4_-ε), 3.18–3.15 (m, 2H, Lys_5_-ε), 3.14–3.05 (m, 36H, methyl), 2.31–2.20 (m, 2H, Aliphatic_1_-a), 2.19–2.13 (m, 2H, Aliphatic_2_-a), 1.97–1.87 (m, 4H, Lys_2,3,4,5_-β), 1.87–1.82 (m, 4H, Lys_2,3,4,5_-β), 1.81–1.67 (m, 10H, Lys_1_-β + Lys_1,2,3,4_-δ), 1.62–1.55 (m, 4H, Aliphatic_1,2_-b), 1.54–1.37 (m, 12H, Lys_1,2,3,4,5_-γ + Lys_5_-δ), 1.34–1.24 (m, 16H, Aliphatic_1,2_-c), 0.94–0.82 (m, 6H, Aliphatic_1,2_-d).

^13^C NMR (126 MHz, MeOD) δ 175.23, 174.89, 174.85, 173.61, 172.69, 172.62, 172.39 (carbonyl), 66.20, 66.13, 66.12, 66.02 (Lys_1,2,3,4_-ε), 53.97 (Lys_5_-α), 53.16, 53.07, 53.04, 52.69 (Lys_1,2,3,4_-α), 52.15 (methyl), 38.53 (Lys_5_-ε), 35.79 (Aliphatic_2_-a), 35.41 (Aliphatic_1_-a), 31.48, 31.45, 31.17, 30.90, 30.81, 30.77, 30.61, 28.94, 28.88, 28.73, 28.69 (Lys_1,2,3,4,5_-β + 4 Aliphatic_1,2_-c), 25.71, 25.56 (Lys_1,2,3,4,5_-δ + 2 Aliphatic_1,2_-b), 23.01, 22.94, 22.38, 22.30, 22.24, 22.22, 22.13, 21.99, 21.95, 21.93, 21.91, 21.84 (Lys_1,2,3,4,5_-γ + 2 Aliphatic_1,2_-c), 12.98, 12.97 (Aliphatic_1,2_-d). 

MS (+TOF) *m*/*z*: calculated for C_58_H_119_N_11_O_7_^4+^ [M+2TFA]^2+^: 653.949, found: 653.942.

### 4.3. Bacterial Strains

Bacterial strains from the American Type Culture Collection (ATCC) include *A. baumannii* ATCC 17978, *E. coli* ATCC 25922, *Staphylococcus aureus* ATCC 29213, methicillin-resistant *S. aureus* (MRSA) ATCC 33592, *Enterococcus faecalis* ATCC 29212, and *Enterococcus faecium* ATCC. Methicillin-resistant *Staphylococcus epidermidis* (MRSE) 61589 was acquired from the Canadian National Intensive Care Unit (CAN-ICU) surveillance study [34]. Clinical isolates *A. baumannii* AB027, *E. coli* 94393, *E. coli* 94474, and methicillin-susceptible *Staphylococcus epidermidis* (MSSE) 81388 were obtained from the Canadian Ward (CAN-WARD) surveillance study [35].

### 4.4. Antimicrobial Susceptibility Assay

The in vitro antibacterial activity of the UTBLPs was assessed against wild-type and clinically isolated bacterial strains. To obtain the MIC of the compounds, the microbroth dilution susceptibility assay was performed according to the Clinical and Laboratory Standards Institute (CLSI, Wayne, PA, USA) guidelines [36] and as previously described [15,33]. Briefly, the compounds at varying concentrations were incubated with bacterial inoculum (5 × 10^5^ CFU/mL final concentration) at 37 °C for 18 h. Growth in the form of turbidity was confirmed using an EMax Plus microplate reader (Molecular Devices, San Jose, CA, USA) at 590 nm.

### 4.5. Checkerboard Assay

The synergy of the UTBLPs with various antibiotics was assessed using checkerboard assay as previously described [15,33]. Briefly, the combination of antibiotic and OMP at varying concentrations were incubated with bacterial inoculum (5 × 10^5^ CFU/mL final concentration) at 37 °C for 18 h. Growth in the form of turbidity was confirmed using an EMax Plus microplate reader (Molecular Devices, Union City, CA, USA) at 590 nm. Fractional inhibitory concentration index (FICI) was used to evaluate the interaction between the antibiotic and the OMP. FICI is the sum of the FIC of the antibiotic and the FIC of the OMP. The FIC value is calculated by dividing the MIC of the agents in combination by the MIC of the agent alone. FICI ≤ 0.5 is synergistic, 0.5 < x ≤ 4 is additive, and >4 is antagonistic [30].

### 4.6. OM Permeabilization Assay

The ability of UTBLPs to permeabilize the OM was assessed using NPN as previously described with minor modifications [13,15,33,37]. In brief, NPN (10 *μ*M final concentration) was incubated with the cell suspension (OD_600_ = 0.4–0.6) in buffer (5 mM HEPES, 5 mM glucose, 5 μM carbonyl cyanide 3-chlorophenylhydrazone, pH 7.2) at room temperature for 30 min in darkness. Varying concentrations of compound diluted in the same buffer were subsequently added to the plate, and the fluorescence (λ_Ex_ = 350 nm, λ_Em_ = 420 nm) was monitored every 30 s on a SpectraMax M2 microplate reader (Molecular Devices, Union City, CA, USA). Measurements were done in triplicates.

## 5. Conclusions

This study demonstrates that an increase in *N*-ζ-methylation of lysine in UTBLPs reduces or abolishes the potentiating effects in these compounds to synergize with OM-impermeable antibiotics like rifampicin, novobiocin, and niclosamide. Our structure–activity relationship results are of interest for the optimization of OMPs that are currently undergoing clinical and preclinical testing as well as for the design of the next generation of OMPs. It is expected that our results can be applied to other classes of related polybasic OMPs including antimicrobial peptides, antimicrobial peptidomimetics, amphiphilic aminoglycosides, and cationic lipids. 

## Figures and Tables

**Table 1:** Antibacterial activity of UTBLPs **1**–**8** against wild-type GNB and wild-type GPB.

Organism	MIC (μg/mL)							
	1	2	3	4	5	6	7	8
*P. aeruginosa* PAO1	>128	>128	>128	>128	>128	>128	>128	>128
*A. baumannii* ATCC 17978	>128	>128	>128	>128	64	64	128	>128
*E. coli* ATCC 25922	>128	>128	>128	>128	64	32	64	>128
*S. aureus* ATCC 29213	64	128	>128	>128	64	128	>128	>128
MRSA ATCC 33592	128	>128	>128	>128	64	>128	>128	>128
*E. faecalis* ATCC 29212	128	>128	>128	>128	32	32	64	128
*E. faecium* ATCC 27270	64	>128	>128	>128	32	64	128	128

## Data Availability

The data presented in this study are available within the article and the Supplementary Material.

## Supplementary Materials

The following supporting information can be downloaded at: https://www.mdpi.com/2079-6382/11/3/335/s1, Table S1: Antibacterial activity of UTBLPs **1**–**8** against MDR GNB and clinically relevant GPB. Table S2: Synergy evaluation of UTBLPs **1**–**8** combined with RIF, NOV, NIC, or CHL against wild-type GNB; Table S3: Synergy evaluation of UTBLPs **1**–**8** combined with RIF or NOV against MDR GNB; Table S4: Synergy evaluation of UTBLPs **1**–**8** combined with CHL against GPB; Figure S1: Dose-dependent increase in fluorescence of NPN in the presence of (A) UTBLP **1**, (B) UTBLP **2**, (C) UTBLP **3**, (D) UTBLP **4**, and PMBN (control) in *E. coli* ATCC 25922; Figure S2: Dose-dependent increase in fluorescence of NPN in the presence of (A) UTBLP **5**, (B) UTBLP **6**, (C) UTBLP **7**, (D) UTBLP **8**, and PMBN (control) in *A. baumannii* ATCC 17978; Figure S3: Dose-dependent increase in fluorescence of NPN in the presence of (A) UTBLP **5**, (B) UTBLP **6**, (C) UTBLP **7**, (D) UTBLP **8**, and PMBN (control) in *E. coli* ATCC 25922; Figure S4: Comparison of UTBLP-induced fluorescence of NPN at 7 μM of (A) UTBLPs **1**–**4** and (B) UTBLPs **5**–**8** in *A. baumannii* ATCC 17978, and 7 μM of (C) UTBLPs **1**–**4** and (D) UTBLPs **5**–**8** in *E. coli* ATCC 25922; NMR spectra (^1^H, ^13^C, COSY, HSQC and HMBC) of UTBLPs **1**–**8**; Table S5: MICs (in *μ*g/mL) of various antibiotics against MDR GNB used in the study.
