# Supplementary material for: Effects of Lysine N-ζ-Methylation in Ultrashort Tetrabasic Lipopeptides (UTBLPs) on the Potentiation of Rifampicin, Novobiocin, and Niclosamide in Gram-Negative Bacteria
Source: Antibiotics (Basel). 2022 Mar 3;11(3):335. doi: 10.3390/antibiotics11030335 (PMC8963254; doi:10.3390/antibiotics11030335)
Supplement: Supplementary file 1 [file antibiotics-11-00335-s001.zip › antibiotics-1599295-supplementary.pdf]

Effects of lysine *N*-ζ-methylation in ultrashort tetrabasic lipopeptides (UTBLPs) on the potentiation of rifampicin, novobiocin, and niclosamide in Gram-negative bacteria

Linus Schweizer, Danyel Ramirez, Frank Schweizer

Table of Contents:

1. Antibacterial activity of UTBLPs **1-8** against MDR GNB and clinically relevant GPB S2

2. Synergy studies of UTBLPs **1-8** against GNB and GPB.. S3-S7

3. OM permeabilization of NPN by UTBLPs **1-8** against wild-type GNB S8-S11

4. NMR spectra (^{1}H, ^{13}C, COSY, HSQC and HMBC) of UTBLPs **1-8** S12-S31

5. Resistance phenotype of MDR GNB...S32

Table S1. Antibacterial activity of UTBLPs **1-8** against MDR GNB and clinically relevant GPB.

Organism	MIC (µg/mL)							
	1	2	3	4	5	6	7	8
P. aeruginosa PA259-96196	>128	>128	>128	>128	128	>128	>128	>128
P. aeruginosa PA262-101856	128	>128	>128	>128	>128	>128	>128	>128
A. baumannii AB027	128	>128	>128	>128	128	>128	128	>128
A. baumannii AB92247	64	>128	>128	>128	32	32	32	128
E. coli 94393	>128	>128	>128	>128	32	64	64	128
E. coli 94474	>128	>128	>128	>128	128	>128	128	>128
E. cloacae 118564	>128	>128	>128	>128	>128	>128	>128	>128
K. pneumoniae 113250	>128	>128	>128	>128	>128	>128	>128	>128
MRSE 61589	32	64	>128	>128	8	16	32	128
MSSE 81388	32	64	>128	>128	16	16	32	128

Table S2. Synergy evaluation of UTBLPs **1-8** combined with RIF, NOV, NIC, or CHL against wild-type GNB.

Organism	UTBLP	Antibiotic	MIC$_{\text{UTBLP}}$ [MIC$_{\text{combo}}$] (µg/mL)	MIC$_{\text{antibiotic}}$ [MIC$_{\text{combo}}$] (µg/mL)	FIC Index	Interpretation
P. aeruginosa PAO1	**1**	Rifampicin	>128 [4]	16 [2]	0.125<x<0.156	Synergy
	**2**		>128 [8]	16 [8]	0.5<x<0.563	Additive
	**3**		>128 [0.25]	16 [16]	1<x<1.002	Additive
	**4**		>128 [0.25]	16 [16]	1<x<1.002	Additive
	**5**		>128 [16]	8 [0.5]	0.0625<x<0.188	Synergy
	**6**		>128 [16]	16 [1]	0.0625<x<0.188	Synergy
	**7**		>128 [16]	8 [4]	0.5<x<0.625	Additive
	**8**		>128 [0.25]	16 [16]	1<x<1.002	Additive
	**1**	Novobiocin	>128 [16]	512 [32]	0.063<x<0.188	Synergy
	**2**		>128 [16]	1024 [256]	0.25<x<0.375	Synergy
	**3**		>128 [16]	1024 [512]	0.5<x<0.675	Additive
	**4**		>128 [16]	1024 [512]	0.5<x<0.675	Additive
	**5**		>128 [16]	512 [8]	0.016<x<0.141	Synergy
	**6**		>128 [16]	512 [32]	0.063<x<0.188	Synergy
	**7**		>128 [16]	512 [128]	0.25<x<0.375	Synergy
	**8**		>128 [0.25]	512 [512]	1<x1.002	Additive
	**1**	Niclosamide	>128 [0.25]	1024 [1024]	1<x<1.002	Additive
	**2**		>128 [0.25]	1024 [1024]	1<x<1.002	Additive
	**3**		>128 [0.25]	1024 [1024]	1<x<1.002	Additive
	**4**		>128 [0.25]	1024 [1024]	1<x<1.002	Additive
	**5**		>128 [4]	1024 [4]	0.004<x<0.035	Synergy
	**6**		>128 [16]	1024 [2]	0.002<x<0.127	Synergy
	**7**		>128 [0.25]	1024 [1024]	1<x<1.002	Additive
	**8**		>128 [0.25]	1024 [1024]	1<x<1.002	Additive
	**1**	Chloramphenicol	>128 [2]	32 [4]	0.125<x<0.141	Synergy
	**2**		>128 [8]	32 [8]	0.25<x<0.313	Synergy
	**3**		>128 [8]	16 [8]	0.5<x<0.563	Additive
	**4**		>128 [16]	16 [16]	1<x<1.002	Additive
	**5**		>128 [2]	16 [2]	0.125<x<0.141	Synergy
	**6**		>128 [16]	16 [2]	0.125<x<0.25	Synergy
	**7**		>128 [8]	16 [8]	0.5<x<0.563	Additive
	**8**		>128 [0.25]	16 [16]	1<x<1.002	Additive
A. baumannii ATCC 17978	**1**	Rifampicin	>128 [8]	2 [0.125]	0.063<x<0.125	Synergy
	**2**		>128 [16]	1 [0.031]	0.031<x<0.156	Synergy
	**3**		>128 [8]	2 [0.5]	0.25<x<0.313	Synergy
	**4**		>128 [16]	1 [0.5]	0.5<x<0.625	Additive
	**5**		64 [1]	2 [0.031]	0.031	Synergy
	**6**		64 [2]	2 [0.031]	0.047	Synergy
	**7**		128 [4]	2 [0.125]	0.094	Synergy
	**8**		>128 [16]	2 [0.25]	0.125<x<0.25	Synergy
	**1**	Novobiocin	>128 [16]	32 [0.125]	0.004<x<0.129	Synergy
	**2**		>128 [8]	32 [0.5]	0.016<x<0.078	Synergy
	**3**		>128 [8]	16 [1]	0.063<x<0.125	Synergy
	**4**		>128 [16]	32 [4]	0.125<x<0.25	Synergy
	**5**		64 [2]	8 [0.125]	0.047	Synergy
	**6**		64 [4]	8 [0.25]	0.063	Synergy
	**7**		128 [4]	16 [0.5]	0.063	Synergy
	**8**		>128 [16]	16 [4]	0.063<x<0.189	Synergy
	**1**	Niclosamide	>128 [8]	512 [0.5]	0.001<x<0.126	Synergy
	**2**		>128 [8]	1024 [2]	0.002<x<0.064	Synergy
	**3**		>128 [0.25]	1024 [1024]	1<x<1.002	Additive
	**4**		>128 [0.25]	1024 [1024]	1<x<1.002	Additive
	**5**		64 [0.5]	>1024 [1]	x<0.009	Synergy
	**6**		64 [1]	>1024 [4]	x<0.020	Synergy
	**7**		128 [4]	>1024 [4]	x<0.035	Synergy
	**8**		>128 [2]	>1024 [16]	x<0.031	Synergy
	**1**	Chloramphenicol	>128 [32]	64 [32]	0.5<x<0.75	Additive
	**2**		>128 [0.25]	128 [64]	0.5<x<0.502	Additive
	**3**		>128 [16]	128 [64]	0.5<x<0.625	Additive
	**4**		>128 [0.25]	128 [128]	1<x<1.002	Additive
	**5**		64 [4]	64 [32]	0.563	Additive
	**6**		64 [8]	64 [32]	0.625	Additive
	**7**		128 [0.25]	64 [64]	1.002	Additive
	**8**		>128 [0.25]	64 [64]	1<x<1.002	Additive
E. coli ATCC 25922	**1**	Rifampicin	>128 [8]	4 [0.125]	0.031<x<0.094	Synergy
	**2**		>128 [16]	4 [0.125]	0.031<x<0.156	Synergy
	**3**		>128 [16]	4 [1]	0.25<x<0.375	Synergy
	**4**		>128 [8]	4 [2]	0.5<x<0.563	Additive
	**5**		64 [2]	2 [0.063]	0.063	Synergy
	**6**		32 [4]	2 [0.063]	0.156	Synergy
	**7**		64 [8]	2 [0.125]	0.188	Synergy
	**8**		>128 [16]	2 [1]	0.5<x<0.625	Additive
	**1**	Novobiocin	>128 [8]	32 [0.25]	0.008<x<0.070	Synergy
	**2**		>128 [8]	64 [1]	0.016<x<0.078	Synergy
	**3**		>128 [16]	64 [4]	0.063<x<0.188	Synergy
	**4**		>128 [16]	64 [16]	0.25<x<0.375	Synergy

	**5**		64 [1]	32 [0.5]	0.031	Synergy
	**6**		32 [2]	32 [0.5]	0.078	Synergy
	**7**		64 [2]	32 [2]	0.094	Synergy
	**8**		>128 [16]	32 [2]	0.063<x<0.188	Synergy
	**1**	Niclosamide	>128 [8]	>128 [0.5]	x<0.133	Synergy
	**2**		>128 [4]	>128 [2]	x<0.133	Synergy
	**3**		>128 [16]	>128 [8]	x<0.188	Synergy
	**4**		>128 [ND]	>128 [ND]	ND	ND
	**5**		64 [0.5]	>256 [2]	0.008<x<0.016	Synergy
	**6**		32 [1]	>256 [2]	0.031<x<0.039	Synergy
	**7**		64 [4]	>256 [1]	0.063<x<0.066	Synergy
	**8**		>128 [8]	>256 [8]	x<0.094	Synergy
	**1**	Chloramphenicol	>128 [32]	4 [1]	0.25<x<0.5	Synergy
	**2**		>128 [0.25]	2 [2]	1<x<1.002	Additive
	**3**		>128 [0.5]	2 [2]	1<x<1.004	Additive
	**4**		>128 [1]	2 [2]	1<x<1.008	Additive
	**5**		64 [16]	8 [0.25]	0.281	Synergy
	**6**		32 [8]	8 [2]	0.5	Synergy
	**7**		64 [16]	4 [0.5]	0.375	Synergy
	**8**		>128 [8]	8 [2]	0.313	Synergy

Table S3. Synergy evaluation of UTBLPs **1-8** combined with RIF or NOV against MDR GNB.

Organism	UTBLP	Antibiotic	MIC_{UTBLP} [MIC_{combo}] (μg/mL)	$MIC_{antibiotic}$ [MIC_{combo}] (μg/mL)	FIC Index	Interpretation
P. aeruginosa PA259-96196	**1**	Rifampicin	>128 [16]	16 [0.5]	0.031<x<0.156	Synergy
	**2**		>128 [16]	16 [4]	0.25<x<0.375	Synergy
	**3**		>128 [16]	16 [16]	1<x<1.002	Additive
	**4**		>128 [0.25]	16 [16]	1<x<1.002	Additive
	**5**		>128 [8]	16 [0.5]	0.0938	Synergy
	**6**		>128 [16]	16 [2]	0.125<x<0.25	Synergy
	**7**		>128 [16]	16 [8]	0.5<x<0.625	Additive
	**8**		>128 [16]	16 [16]	1<x<1.002	Additive
	**1**	Novobiocin	>128 [8]	2048 [128]	0.063<x<0.125	Synergy
	**2**		>128 [16]	2048 [512]	0.25<x<0.375	Synergy
	**3**		>128 [0.25]	2048 [2048]	1<x<1.002	Additive
	**4**		>128 [0.25]	2048 [2048]	1<x<1.002	Additive
	**5**		128 [4]	1024 [16]	0.094	Synergy
	**6**		>128 [16]	1024 [128]	0.125<x<0.25	Synergy
	**7**		>128 [16]	1024 [512]	0.5<x<0.625	Additive
	**8**		>128 [16]	1024 [1024]	1<x<1.002	Additive
P. aeruginosa PA262-101856	**1**	Rifampicin	128 [16]	1024 [256]	0.375	Synergy
	**2**		>128 [0.25]	512 [512]	1<x<1.002	Additive
	**3**		>128 [0.25]	512 [512]	1<x<1.002	Additive
	**4**		>128 [0.25]	512 [512]	1<x<1.002	Additive
	**5**		>128 [16]	512 [32]	0.063<x<0.125	Synergy
	**6**		>128 [16]	512 [256]	0.5<x<0.625	Additive
	**7**		>128 [16]	512 [512]	1<x<1.002	Additive
	**8**		>128 [16]	512 [512]	1<x<1.002	Additive
	**1**	Novobiocin	128 [16]	>1024 [64]	0.125<x<0.188	Synergy
	**2**		>128 [16]	1024 [512]	0.5<x<0.625	Additive
	**3**		>128 [8]	2048 [1024]	0.5<x<0.563	Additive
	**4**		>128 [16]	1024 [1024]	1<x<1.125	Additive
	**5**		>128 [16]	1024 [16]	0.016<x<0.141	Synergy
	**6**		>128 [16]	1024 [128]	0.125<x<0.5	Synergy
	**7**		>128 [16]	1024 [512]	0.5<x<0.625	Additive
	**8**		>128 [16]	1024 [1024]	1<x<1.002	Additive
A. baumannii AB027	**1**	Rifampicin	128 [8]	1 [0.063]	0.125	Synergy
	**2**		>128 [16]	1 [0.063]	0.063<x<0.188	Synergy
	**3**		>128 [4[	1 [0.5]	0.5<x<0.531	Additive
	**4**		>128 [0.25]	1 [1]	1<x<1.002	Additive
	**5**		128 [2]	1 [0.016]	0.031	Synergy
	**6**		>128 [16]	1 [0.002]	0.002<x<0.127	Synergy
	**7**		128 [8]	1 [0.063]	0.125	Synergy
	**8**		>128 [16]	1 [0.25]	0.25<x<0.375	Synergy
	**1**	Novobiocin	128 [8]	8 [0.125]	0.078	Synergy
	**2**		>128 [16]	16 [0.25]	0.125<x<0.141	Synergy
	**3**		>128 [16]	16 [2]	0.125<x<0.25	Synergy
	**4**		>128 [16]	16 [4]	0.25<x<0.375	Synergy
	**5**		128]1]	16 [0.25]	0.023	Synergy
	**6**		>128 [16]	16 [0.031]	0.002<x<0.127	Synergy
	**7**		128 [4]	16 [0.5]	0.063	Synergy
	**8**		>128 [16]	16 [2]	0.125<x<0.25	Synergy
A. baumannii 92247	**1**	Rifampicin	64 [4]	1 [0.125]	0.188	Synergy
	**2**		>128 [16]	2 [0.063]	0.031<x<0.156	Synergy
	**3**		>128 [16]	1 [0.25]	0.25<x<0.375	Synergy
	**4**		>128 [0.25]	1 [1]	1<x<1.002	Additive
	**5**		32 [2]	1 [0.063]	0.125	Synergy
	**6**		32 [0.5]	1 [0.063]	0.078	Synergy
	**7**		32 [8]	1 [0.125]	0.375	Synergy
	**8**		128 [16]	1 [0.5]	0.063	Synergy
	**1**	Novobiocin	64 [4]	2 [0.25]	0.188	Synergy
	**2**		>128 [16]	4 [0.25]	0.063<x<0.188	Synergy
	**3**		>128 [8]	4 [1]	0.25<x<0.313	Synergy
	**4**		>128 [1]	4 [4]	1<x<1.008	Additive
	**5**		32 [1]	4 [0.125]	0.063	Synergy
	**6**		32 [1]	2 [0.125]	0.094	Synergy
	**7**		32 [1]	2 [0.25]	0.156	Synergy
	**8**		128 [16]	2 [0.125]	0.188	Synergy
E. coli 94393	**1**	Rifampicin	>128 [8]	4 [0.031]	0.004<x<0.066	Synergy
	**2**		>128 [16]	4 [0.125]	0.031<x<0.156	Synergy
	**3**		>128 [16]	4 [1]	0.25<x<0.375	Synergy
	**4**		>128 [0.25]	4 [4]	1<x<1.002	Additive
	**5**		32 [4]	4 [0.125]	0.156	Synergy
	**6**		64 [4]	4 [0.25]	0.125	Synergy
	**7**		64 [8]	4 [0.5]	0.25	Synergy
	**8**		128 [16]	4 [20	0.625	Additive
	**1**	Novobiocin	>128 [8]	128 [0.5]	0.004<x<0.066	Synergy
	**2**		>128 [16]	64 [1]	0.016<x<0.141	Synergy
	**3**		>128 [16]	64 [8]	0.125<x<0.25	Synergy
	**4**		>128 [0.25]	64 [32]	0.5<x<0.502	Additive
	**5**		32 [1]	64 [2]	0.063	Synergy
	**6**		64 [4]	64 [1]	0.078	Synergy

	**7**		64 [4]	64 [2]	0.093	Synergy
	**8**		128 [16]	64 [16]	0.375	Synergy
E. coli 94474	**1**	Rifampicin	>128 [8]	4 [0.125]	0.031<x<0.094	Synergy
	**2**		>128 [16]	8 [0.5]	0.063<x<0.188	Synergy
	**3**		>128 [16]	4 [2]	0.5<x<0.625	Additive
	**4**		>128 [16]	4 [4]	1<x<1.002	Additive
	**5**		128 [2]	8 [0.25]	0.047	Synergy
	**6**		>128 [16]	4 [0.125]	0.031<x<0.156	Synergy
	**7**		128 [16]	4 [1]	0.375	Synergy
	**8**		>128 [16]	8 [4]	0.5<x<0.625	Additive
	**1**	Novobiocin	>128 [8]	256 [4]	0.016<x<0.078	Synergy
	**2**		>128 [16]	256 [4]	0.016<x<0.141	Synergy
	**3**		>128 [16]	256 [32]	0.125<x<0.25	Synergy
	**4**		>128 [16]	256 [128]	0.5<x<0.625	Additive
	**5**		128 [4]	128 [1]	0.039	Synergy
	**6**		>128 [16]	128 [1]	0.008<x<0.133	Synergy
	**7**		128 [8]	256 [16]	0.125	Synergy
	**8**		>128 [8]	256 [64]	0.25<x<0.313	Synergy
E. cloacae 118564	**1**	Rifampicin	>128 [8]	8 [0.125]	0.016<x<0.078	Synergy
	**2**		>128 [16]	8 [0.2]	0.031<x<0.156	Synergy
	**3**		>128 [8]	8 [2]	0.25<x<0.313	Synergy
	**4**		>128 [16]	8 [4]	0.5<x<0.625	Additive
	**5**		>128 [8]	8 [0.5]	0.063<x<0.125	Synergy
	**6**		>128 [16]	8 [0.5]	0.063<x<0.188	Synergy
	**7**		>128 [16]	8 [1]	0.125<x<0.25	Synergy
	**8**		>128 [16]	8 [4]	0.5<x<0.563	Additive
	**1**	Novobiocin	>128 [8]	256 [2]	0.008<x<0.070	Synergy
	**2**		>128 [16]	256 [8]	0.031<x<0.094	Synergy
	**3**		>128 [16]	256 [16]	0.063<x<0.188	Synergy
	**4**		>128 [16]	256 [128]	0.5<x<0.563	Additive
	**5**		>128 [16]	256 [0.5]	0.002<x<0.127	Synergy
	**6**		>128 [16]	256 [1]	0.004<x<0.129	Synergy
	**7**		>128 [16]	256 [4]	0.016<x<0.141	Synergy
	**8**		>128 [16]	256 [32]	0.125<x<0.25	Synergy
K. pneumoniae 113250	**1**	Rifampicin	>128 [16]	16 [0.5]	0.031<x<0.156	Synergy
	**2**		>128 [16]	16 [4]	0.125<x<0.188	Synergy
	**3**		>128 [16]	16 [4]	0.25<0.313	Synergy
	**4**		>128 [0.25]	8 [8]	1<x<1.002	Additive
	**5**		>128 [8]	8 [0.5]	0.063<x<0.125	Synergy
	**6**		>128 [16]	8 [1]	0.125<x<0.25	Synergy
	**7**		>128 [16]	8 [2]	0.25<x<0.375	Synergy
	**8**		>128 [16]	16 [4]	0.25<x<0.375	Synergy
	**1**	Novobiocin	>128 [8]	256 [16]	0.063<x<0.094	Synergy
	**2**		>128 [16]	256 [4]	0.016<x<0.141	Synergy
	**3**		>128 [16]	256 [32]	0.125<x<0.25	Synergy
	**4**		>128 [16]	256 [128]	0.5<x<0.625	Additive
	**5**		>128 [16]	64 [1]	0.016<x<0.141	Synergy
	**6**		>128 [16]	64 [2]	0.031<x<0.156	Synergy
	**7**		>128 [8]	128 [8]	0.063<x<0.125	Synergy
	**8**		>128 [16]	128 [32]	0.25<x<0.375	Synergy

Table S4. Synergy evaluation of UTBLPs **1-8** combined with CHL against GPB.

Organism	UTBLP	MIC_{UTBLP} [MIC_{combo}] (μg/mL)	$MIC_{chloramphenicol}$ [MIC_{combo}] (μg/mL)	FIC Index	Interpretation
S. aureus ATCC 29213	**1**	64 [0.25]	8 [8]	1.004	Additive
	**2**	128 [0.25]	16 [16]	1.002	Additive
	**3**	>128 [0.25]	16 [16]	1<x<1.002	Additive
	**4**	>128 [0.25]	16 [16]	1<x<1.002	Additive
	**5**	64 [16]	8 [2]	0.5	Additive
	**6**	128 [16]	8 [4]	0.625	Additive
	**7**	>128 [0.25]	8 [8]	1<x<1.002	Additive
	**8**	>128 [0.25]	8 [8]	1<x<1.002	Additive
MRSA ATCC 33592	**1**	128 [0.25]	64 [64]	1.002	Additive
	**2**	>128 [0.25]	64 [64]	1<x<1.002	Additive
	**3**	>128 [0.25]	64 [64]	1<x<1.002	Additive
	**4**	>128 [0.25]	64 [64]	1<x<1.002	Additive
	**5**	64 [0.25]	64 [64]	1<x<1.004	Additive
	**6**	>128 [0.25]	64 [64]	1<x<1.002	Additive
	**7**	>128 [0.25]	64 [64]	1<x<1.002	Additive
	**8**	>128 [0.25]	64 [64]	1<x<1.002	Additive
MRSE 61589	**1**	32 [16]	8 [2]	0.75	Additive
	**2**	64 [0.25]	16 [16]	1.004	Additive
	**3**	>128 [0.25]	16 [16]	1<x<1.002	Additive
	**4**	>128 [0.25]	16 [16]	1<x<1.002	Additive
	**5**	8 [4]	8 [4]	1	Additive
	**6**	16 [8]	8 [4]	1	Additive
	**7**	32 [0.25]	8 [8]	1.008	Additive
	**8**	128 [0.25]	8 [8]	1.002	Additive
MSSE 81388	**1**	32 [0.25]	4 [4]	1.008	Additive
	**2**	64 [0.25]	8 [8]	1.004	Additive
	**3**	>128 [0.25]	8 [8]	1<x<1.002	Additive
	**4**	>128 [0.25]	8 [8]	1<x<1.002	Additive
	**5**	16 [8]	4 [0.25]	1.063	Additive
	**6**	16 [0.25]	4 [4]	1.016	Additive
	**7**	32 [0.25]	4 [4]	1.008	Additive
	**8**	128 [0.25]	4 [4]	1.002	Additive
E. faecalis ATCC 29212	**1**	128 [16]	4 [2]	0.625	Additive
	**2**	>128 [0.25]	8 [8]	1<x<1.002	Additive
	**3**	>128 [0.25]	8 [8]	1<x<1.002	Additive
	**4**	>128 [0.25]	8 [8]	1<x<1.002	Additive
	**5**	32 [4]	8 [4]	0.625	Additive
	**6**	32 [4]	8 [4]	0.625	Additive
	**7**	64 [8]	8 [4]	0.625	Additive
	**8**	128 [16]	8 [4]	0.625	Additive
E. faecium ATCC 27270	**1**	64 [0.25]	8 [8]	1.004	Additive
	**2**	>128 [0.25]	8 [8]	1<x<1.002	Additive
	**3**	>128 [0.25]	8 [8]	1<x<1.002	Additive
	**4**	>128 [0.25]	8 [8]	1<x<1.002	Additive
	**5**	32 [8]	4 [2]	0.75	Additive
	**6**	64 [4]	4 [4]	1.004	Additive
	**7**	128 [0.25]	4 [4]	1.002	Additive
	**8**	>128 [0.25]	4 [4]	1<x<1.002	Additive

Figure S1. Dose-dependent increase in fluorescence of NPN in the presence of (**A**) UTBLP **1**, (**B**) UTBLP **2**, (**C**) UTBLP **3**, (**D**) UTBLP **4**, and PMBN (control) in *E. coli* ATCC 25922.

A

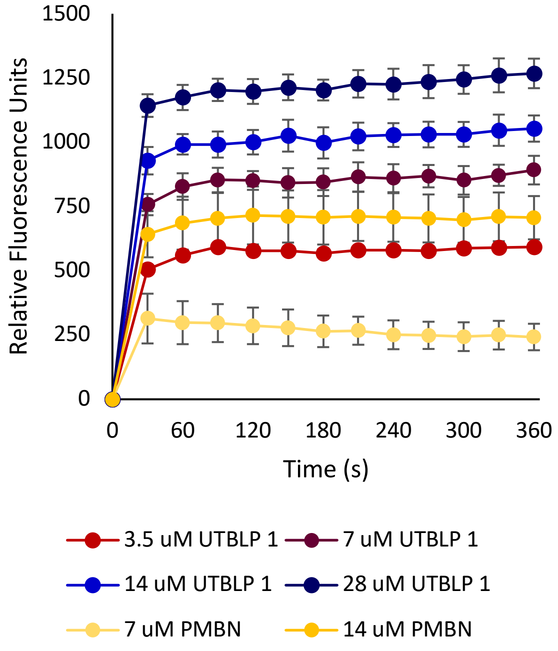

B

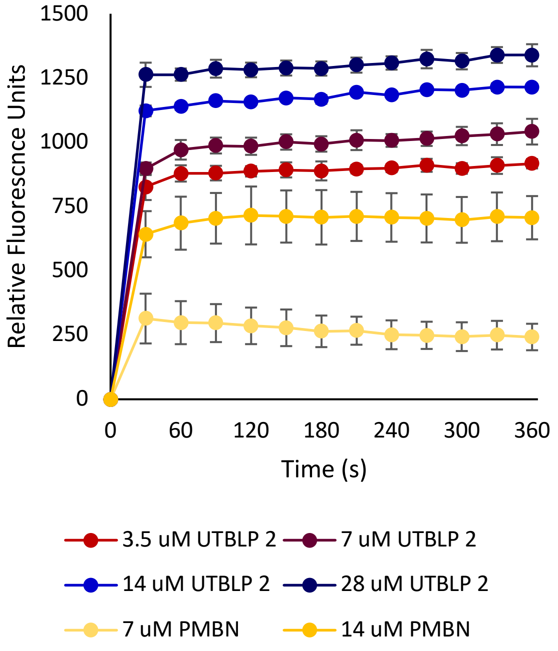

C

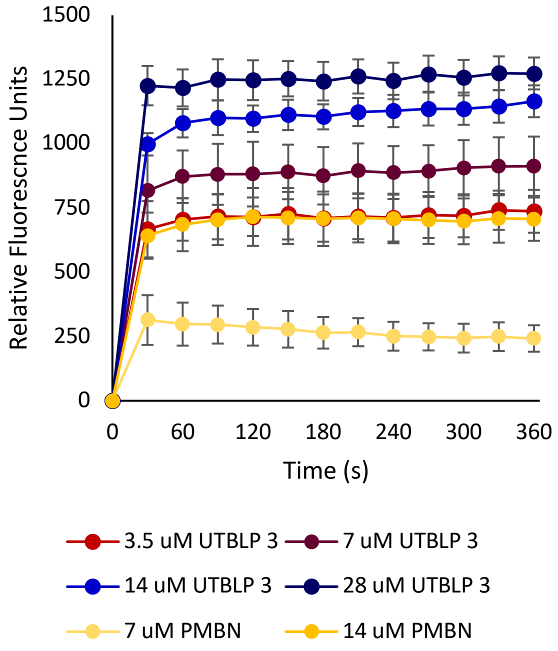

D

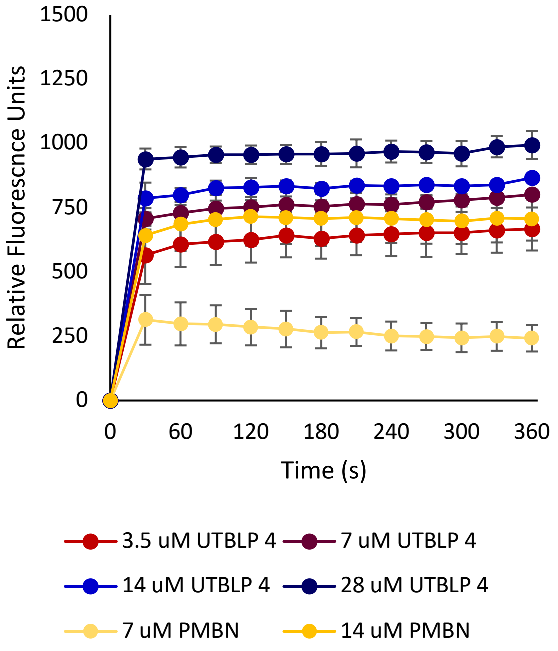

Figure S2. Dose-dependent increase in fluorescence of NPN in the presence of (**A**) UTBLP **5**, (**B**) UTBLP **6**, (**C**) UTBLP **7**, (**D**) UTBLP **8**, and PMBN (control) in *A. baumannii* ATCC 17978.

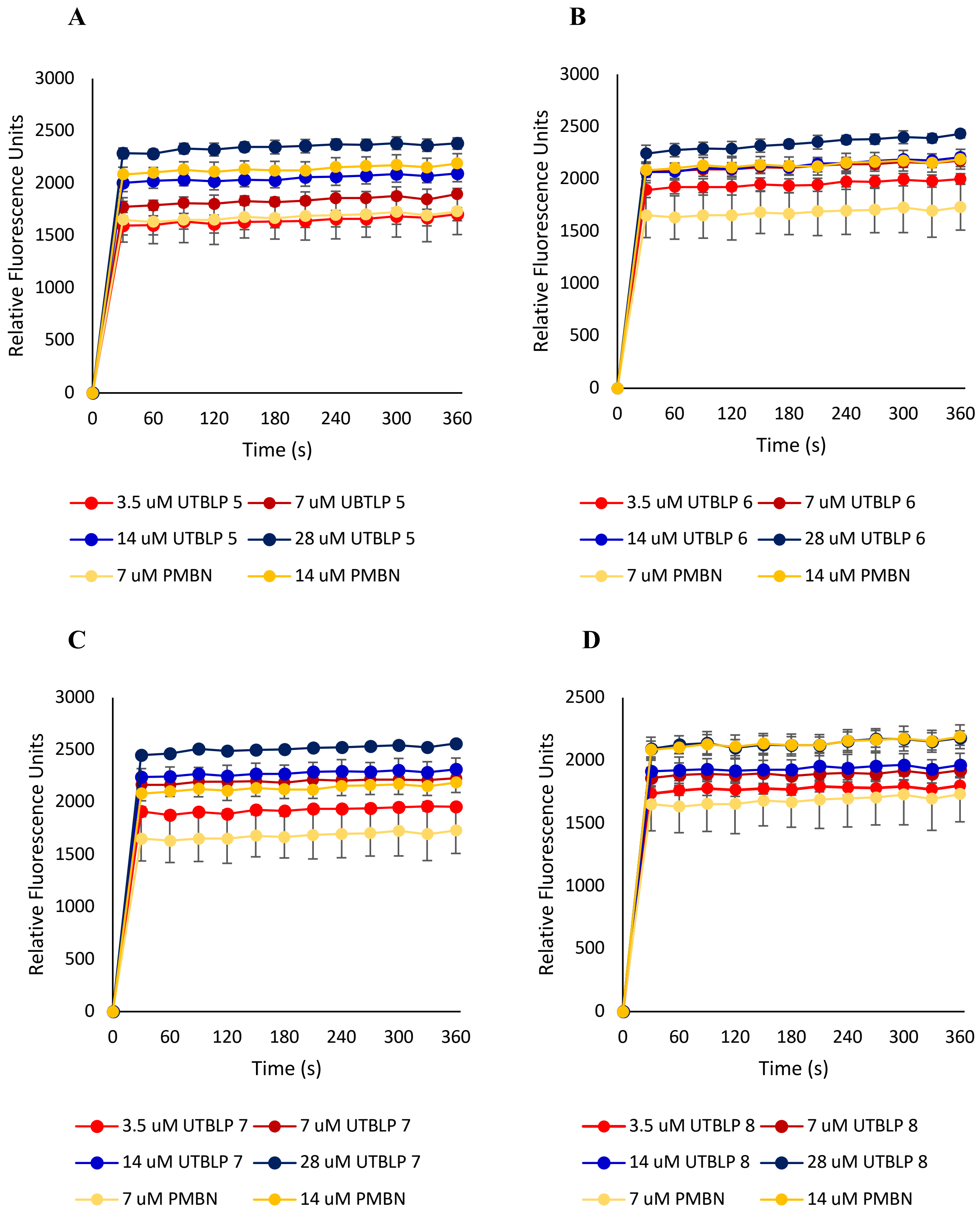

Figure S3. Dose-dependent increase in fluorescence of NPN in the presence of (**A**) UTBLP **5**, (**B**) UTBLP **6**, (**C**) UTBLP **7**, (**D**) UTBLP **8**, and PMBN (control) in *E. coli* ATCC 25922.

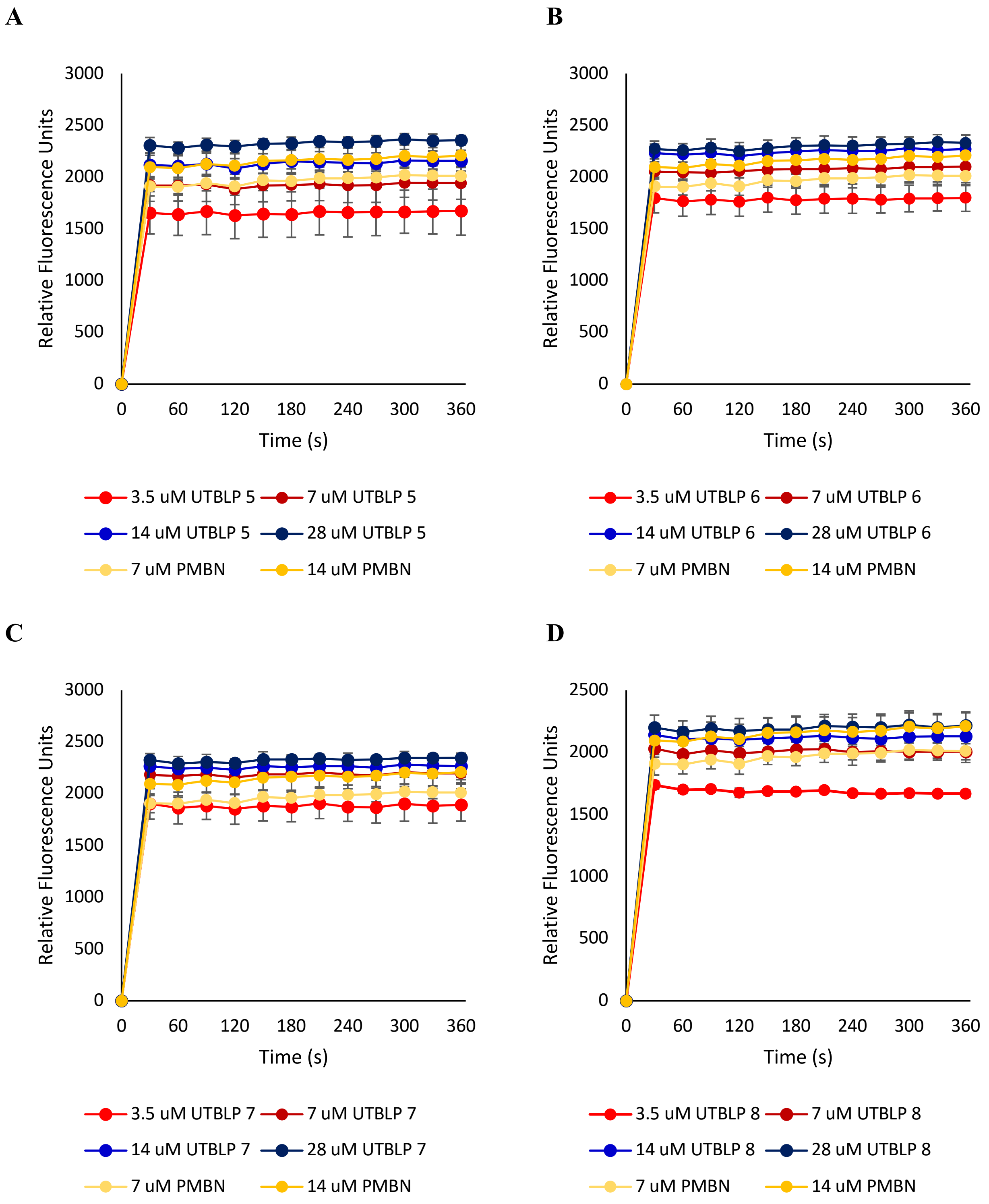

Figure S4. Comparison of UTBLP-induced fluorescence of NPN at 7 μM of (**A**) UTBLPs **1-4** and (**B**) UTBLPs **5-8** in *A. baumannii* ATCC 17978, and 7 μM of (**C**) UTBLPs **1-4** and (**D**) UTBLPs **5-8** in *E. coli* ATCC 25922.

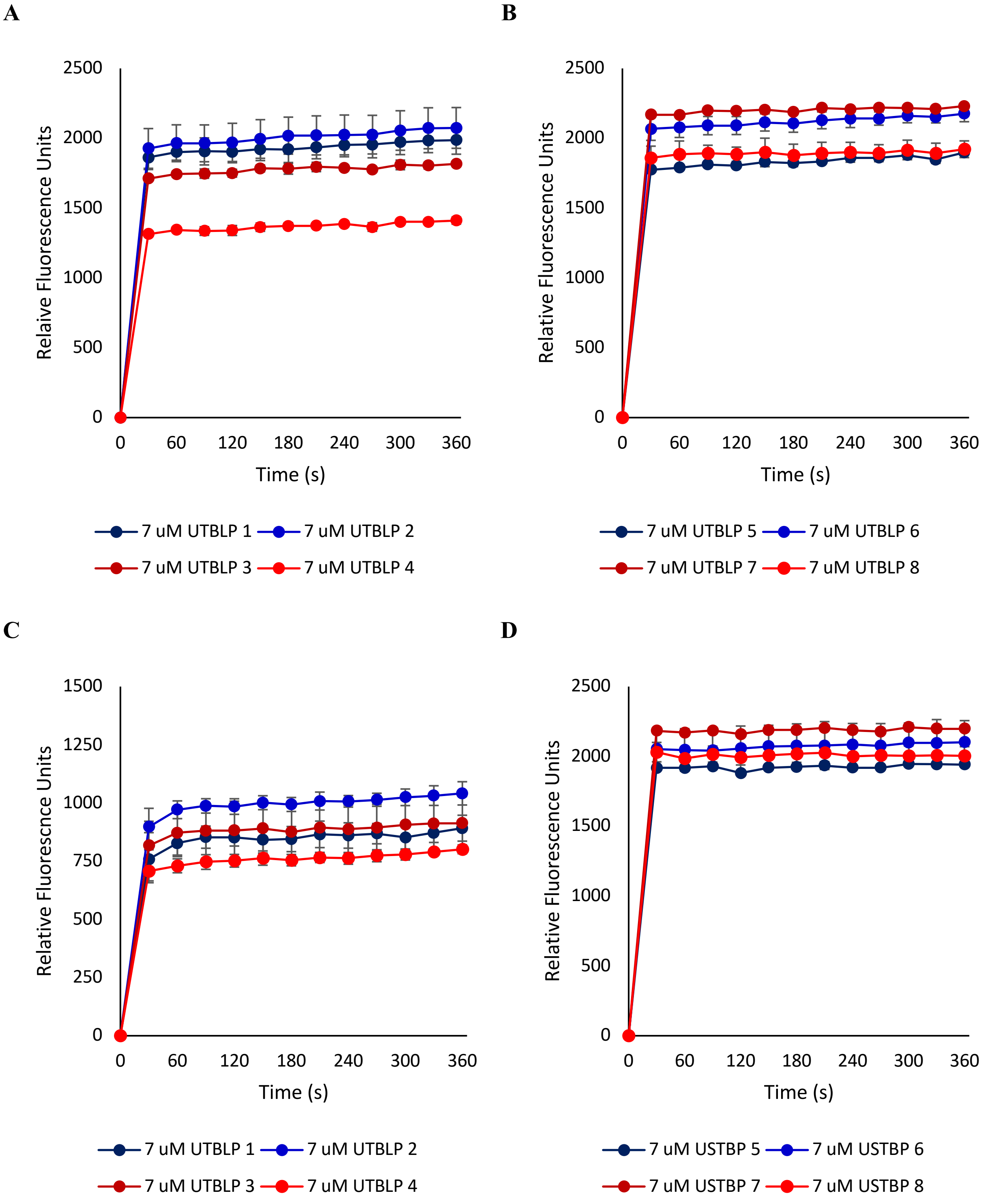

NMR Spectra (^{1}H, ^{13}C, COSY, HSQC and HMBC) of UTBLPs 1-8

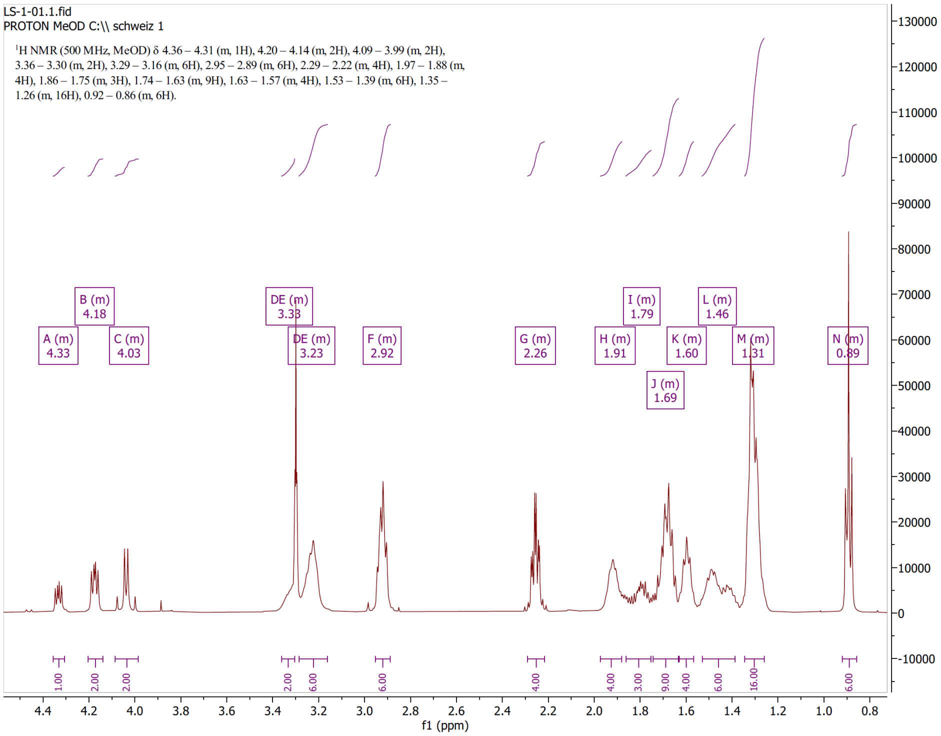

^{1}H spectrum of UTBLP **1**.

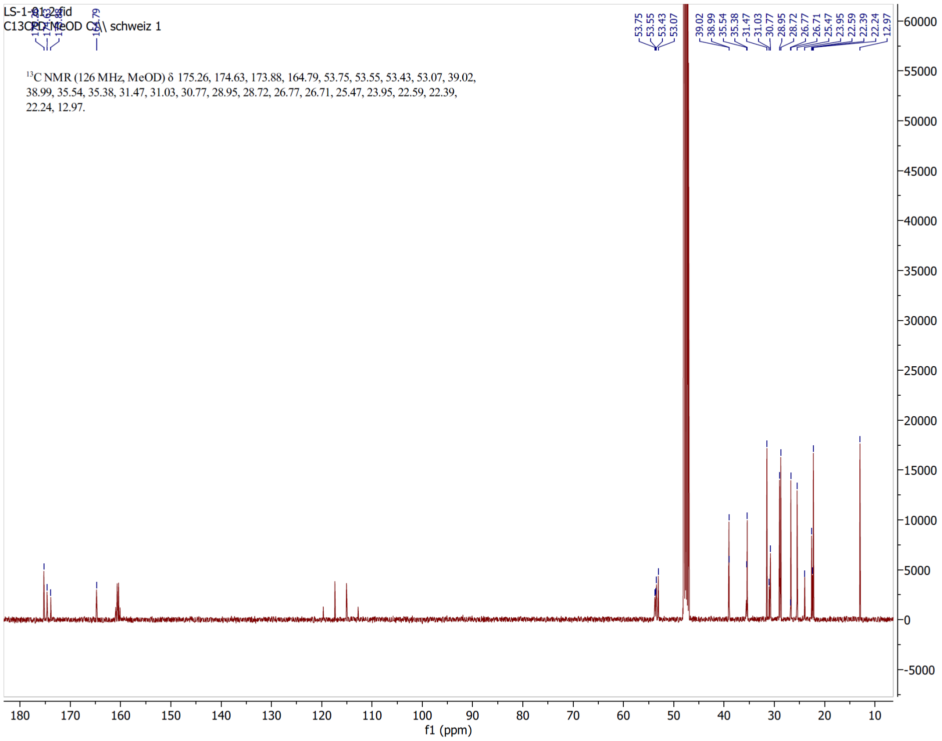

^{13}C spectrum of UTBLP **1**.

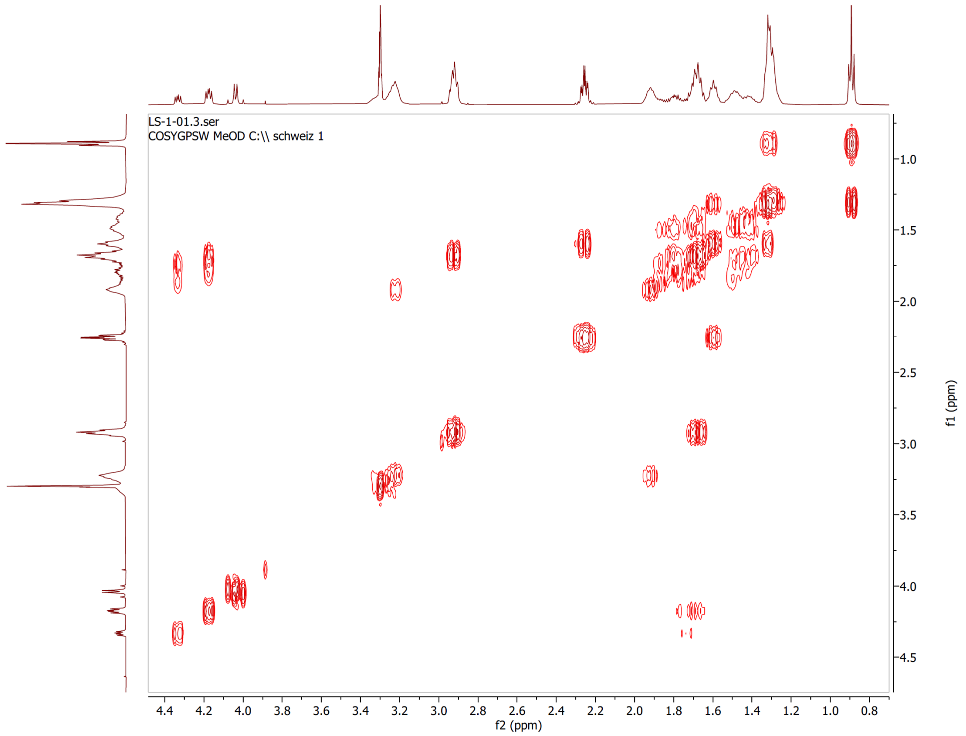

COSY spectrum of UTBLP **1**.

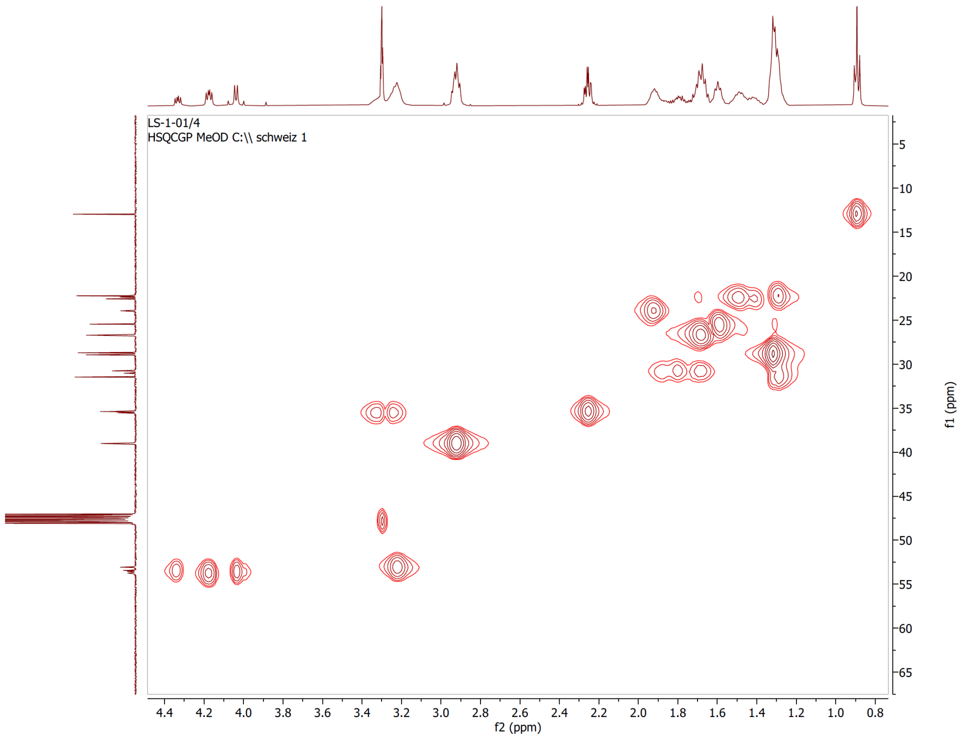

HSQC spectrum of UTBLP **1**.

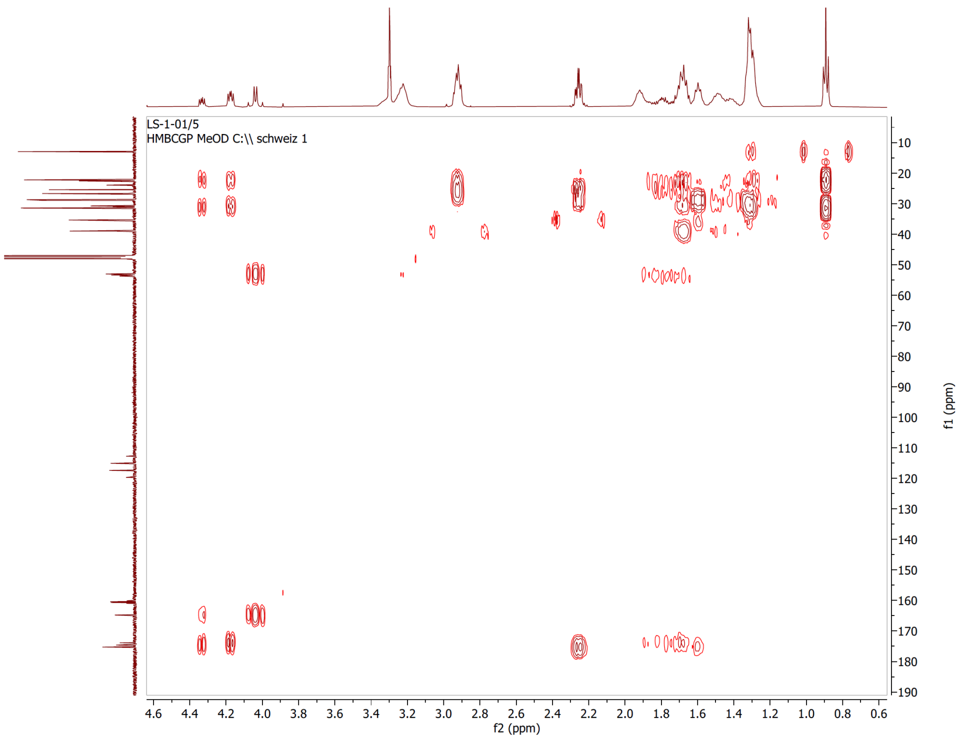

HMBC spectrum of UTBLP **1**.

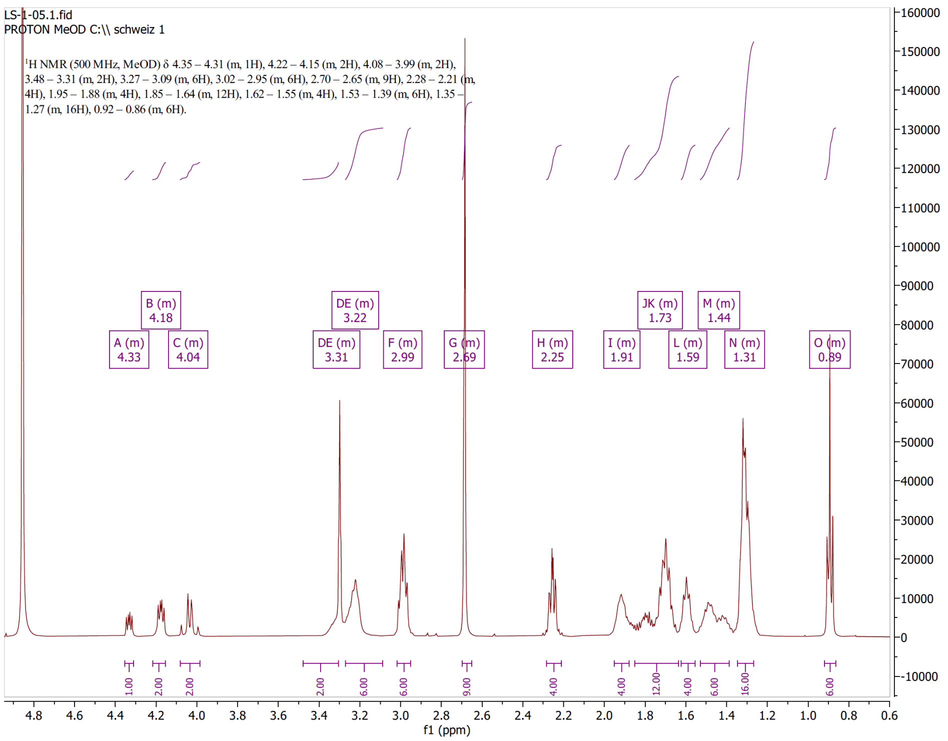

^{1}H spectrum of UTBLP **2**.

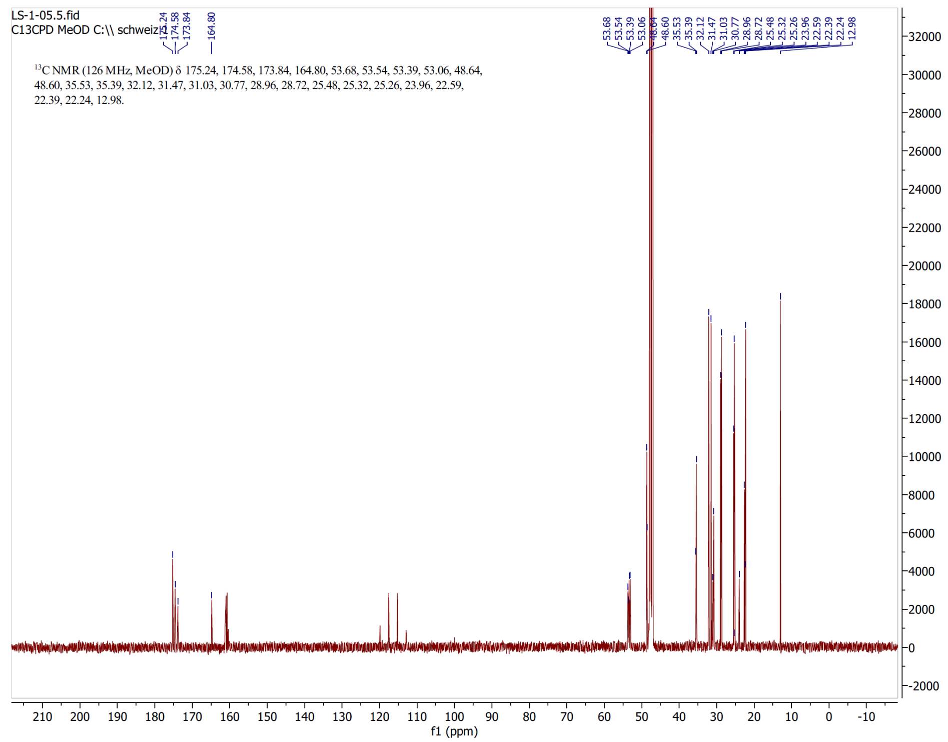

^{13}C spectrum of UTBLP **2**.

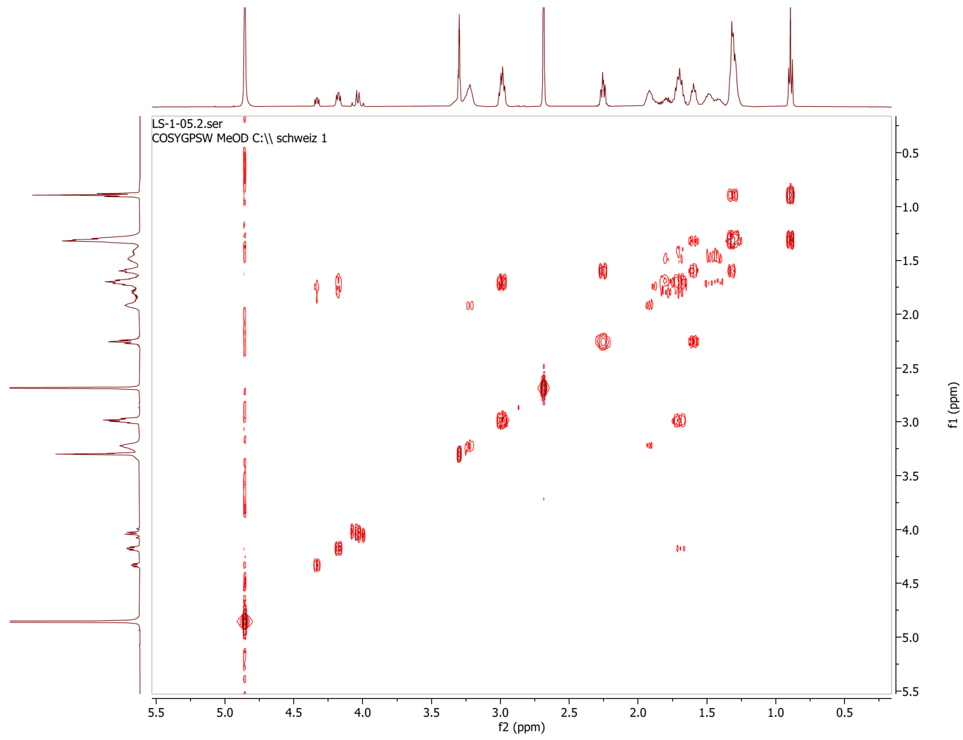

COSY spectrum of UTBLP **2**.

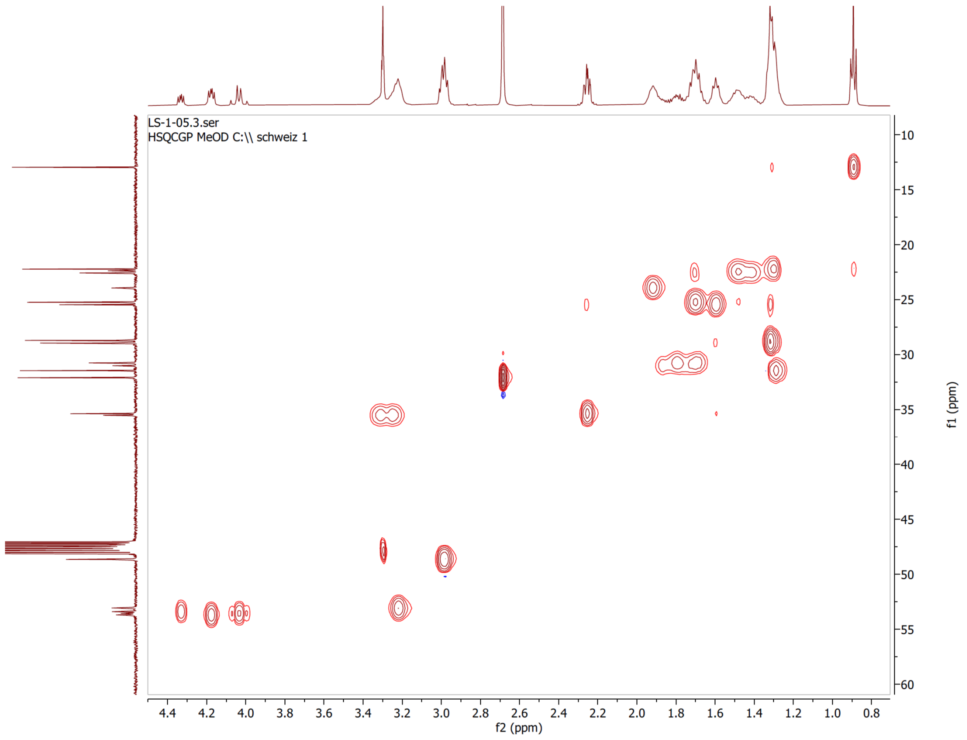

HSQC spectrum of UTBLP **2**.

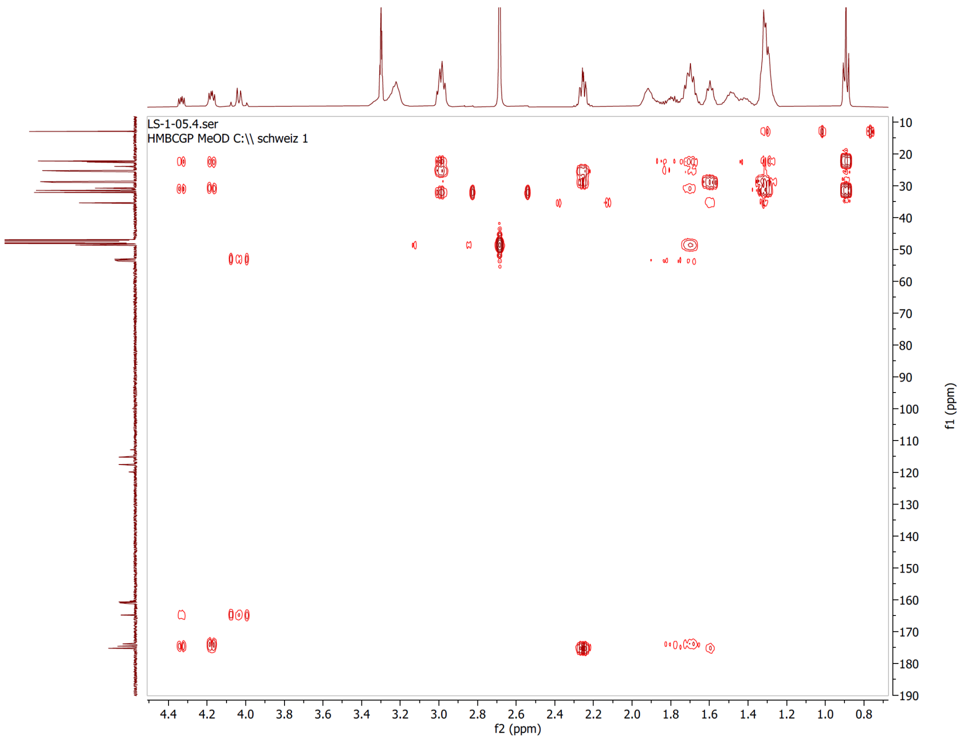

HMBC spectrum of UTBLP **2**.

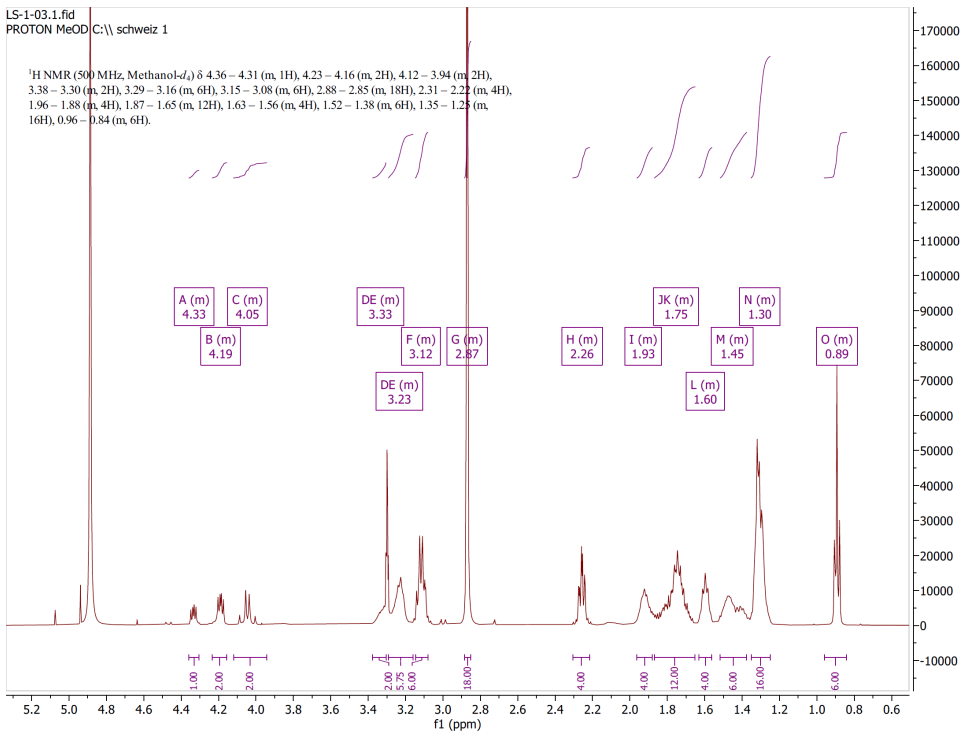

^{1}H spectrum of UTBLP **3**.

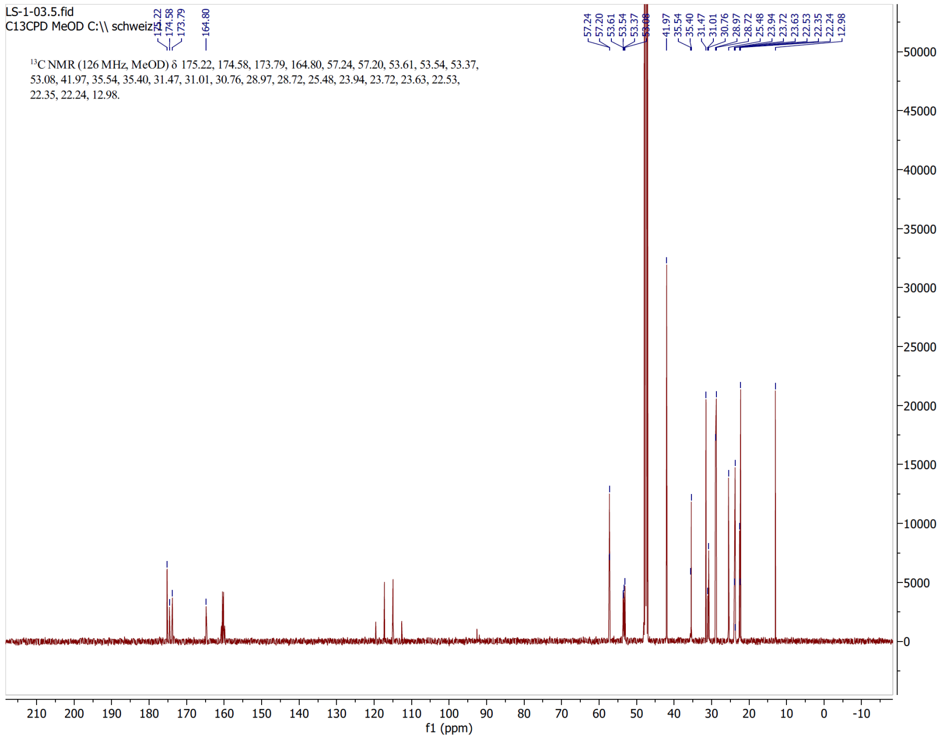

^{13}C spectrum of UTBLP **3**.

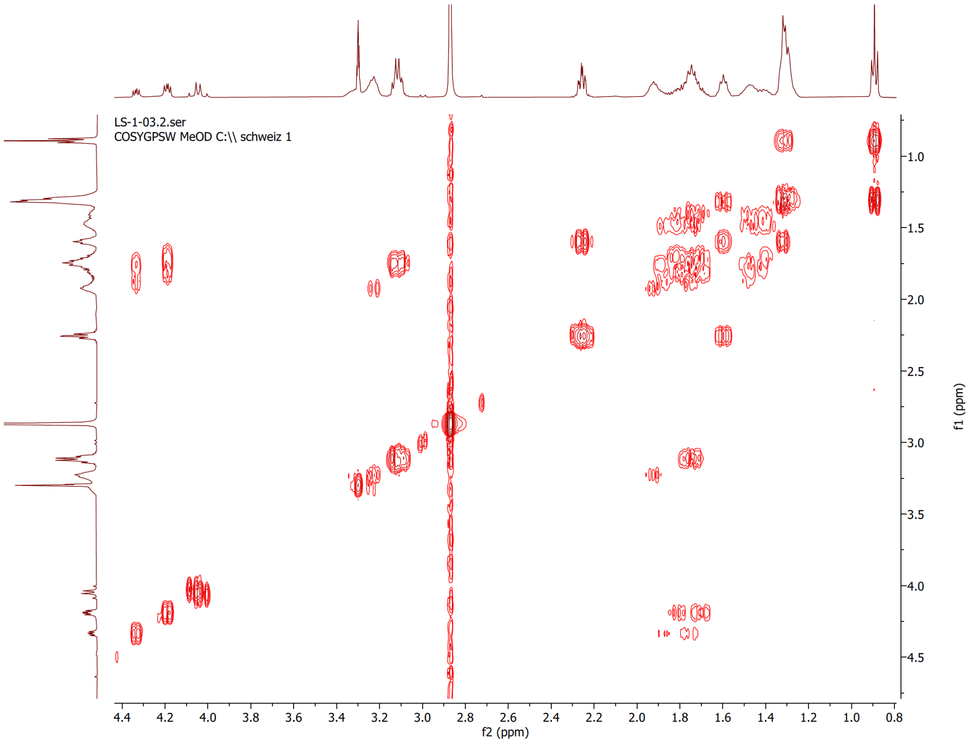

COSY spectrum of UTBLP **3**.

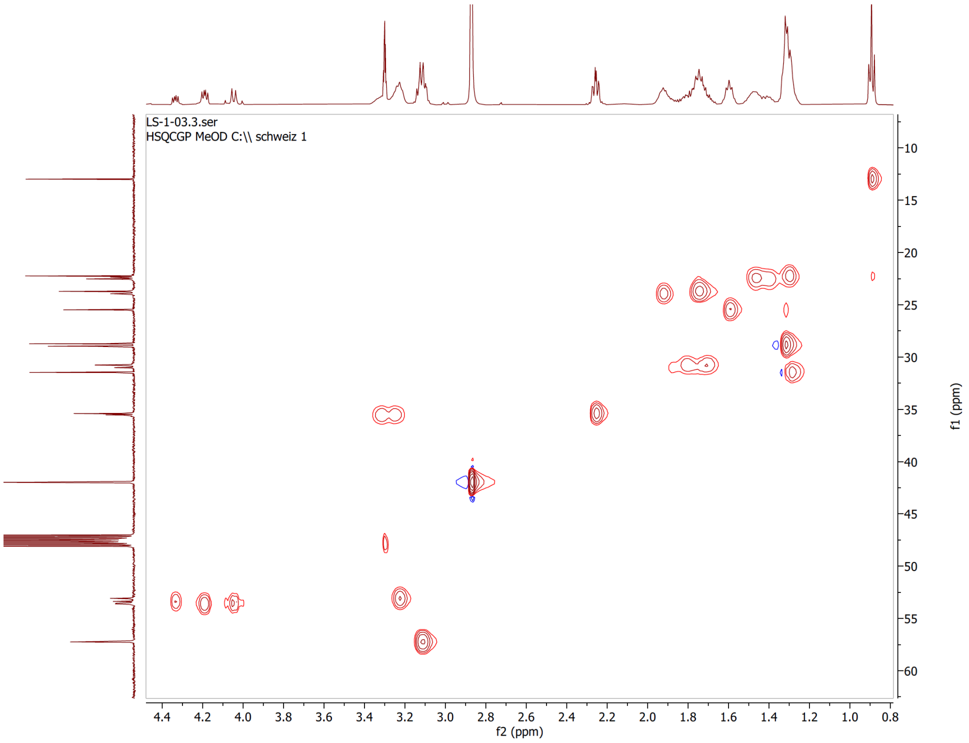

HSQC spectrum of UTBLP **3**.

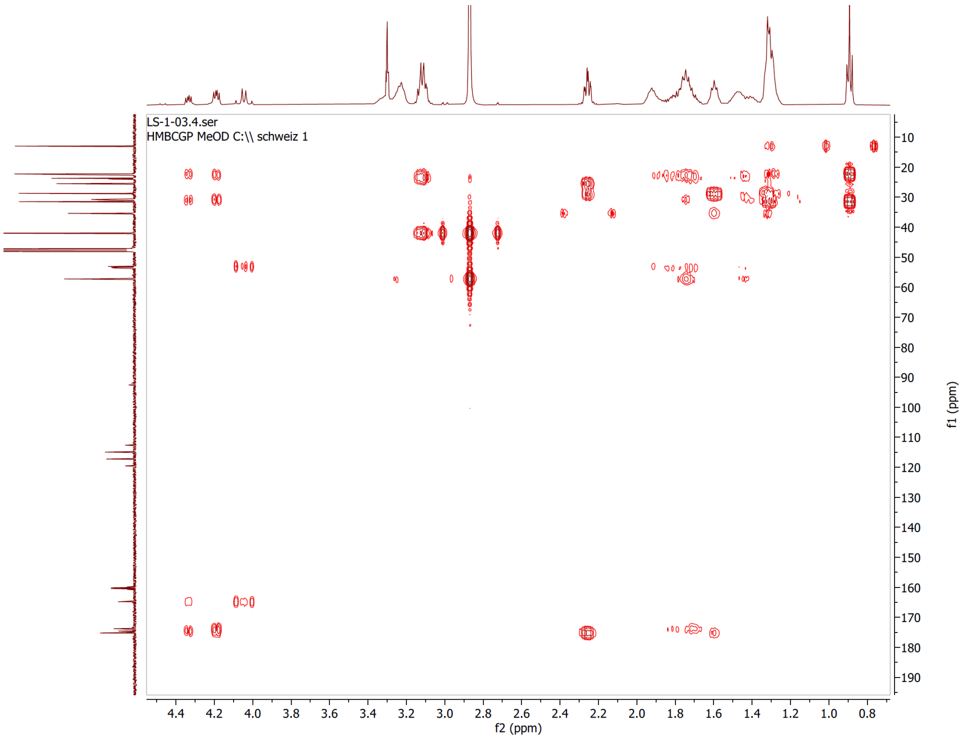

HMBC spectrum of UTBLP **3**.

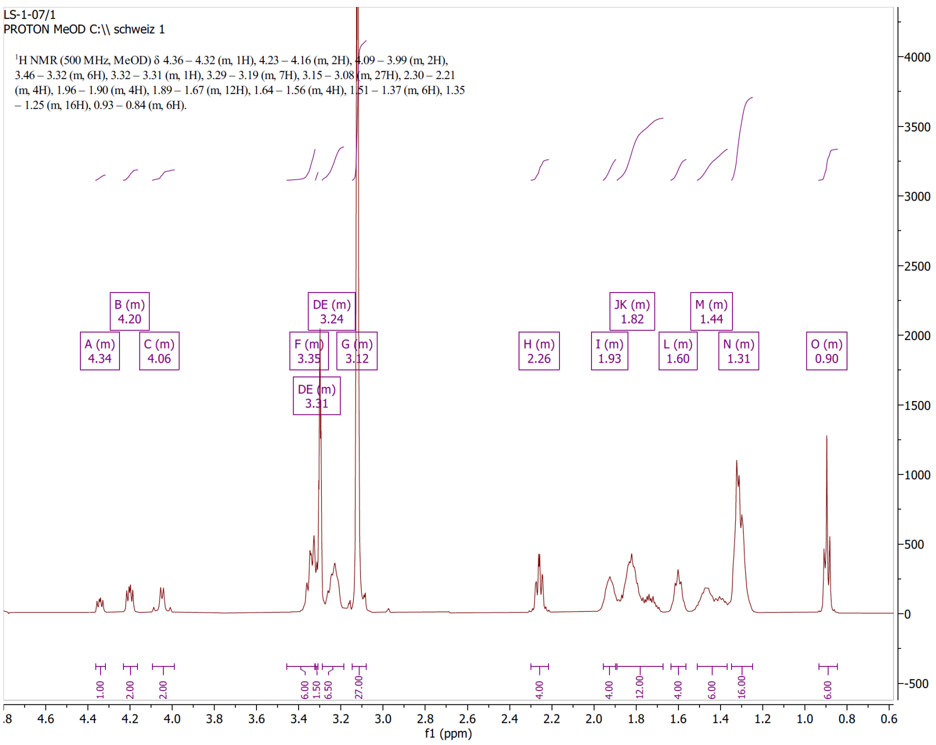

^{1}H spectrum of UTBLP **4**.

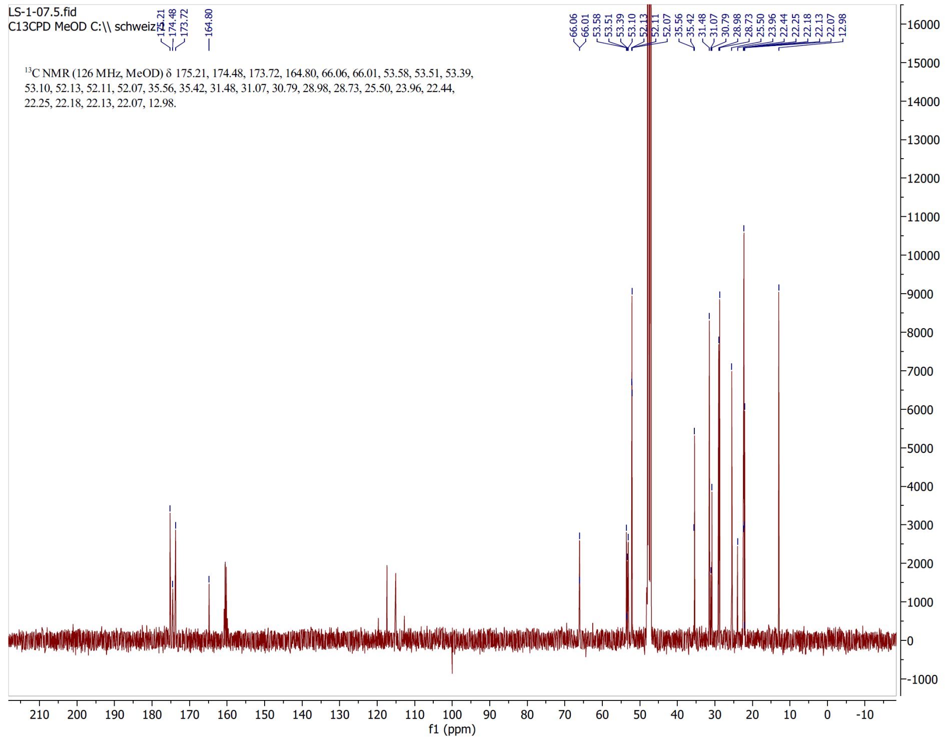

^{13}C spectrum of UTBLP **4**.

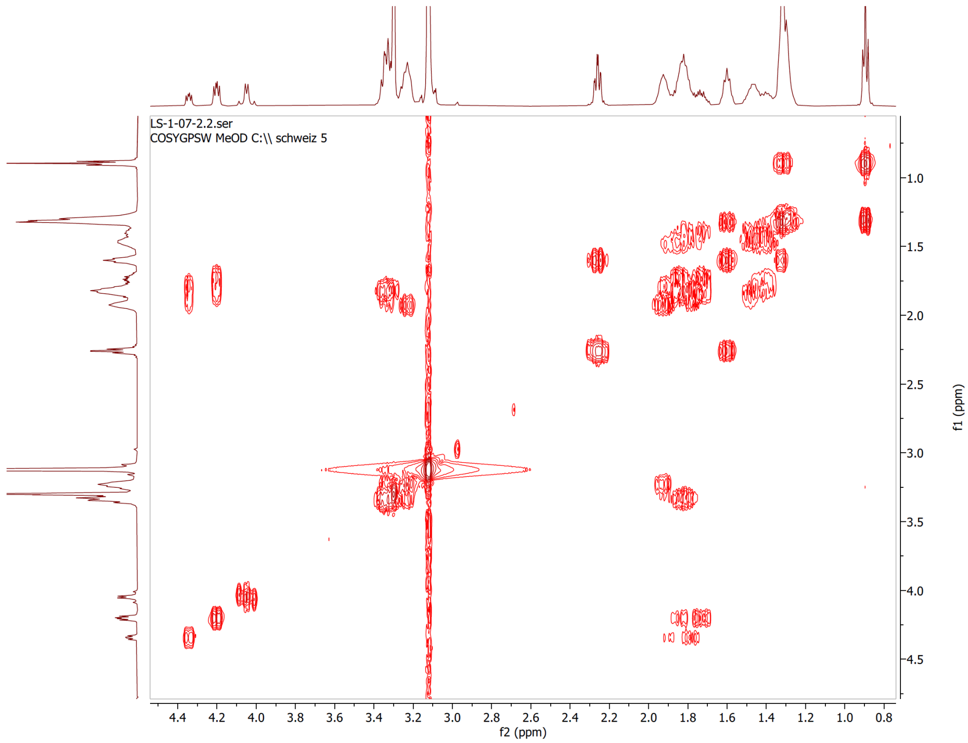

COSY spectrum of UTBLP **4**.

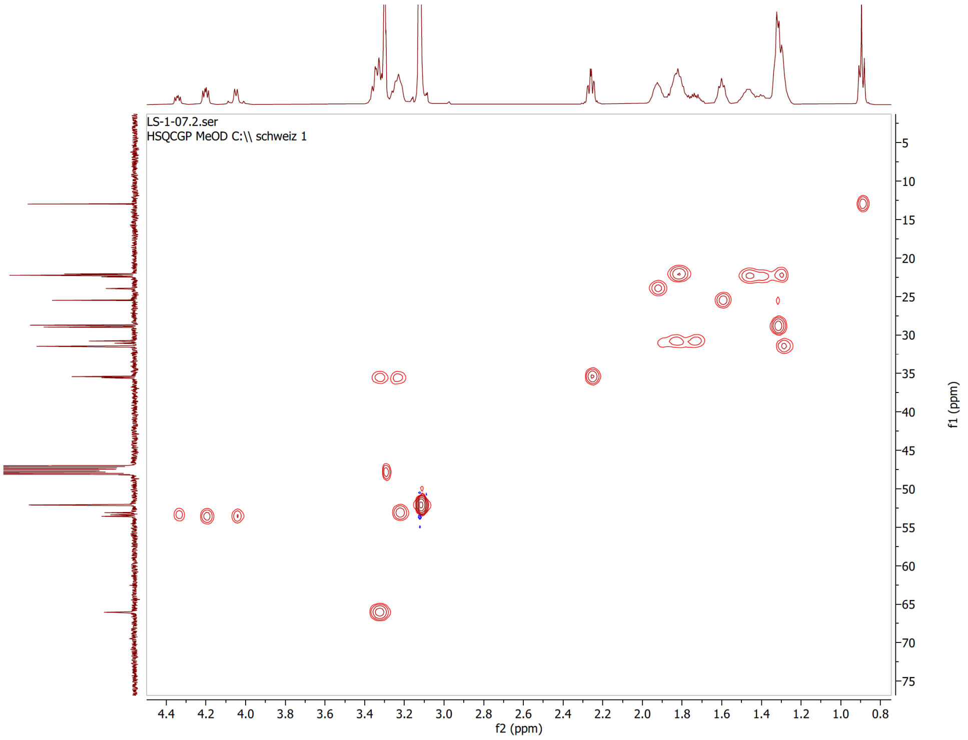

HSQC spectrum of UTBLP **4**.

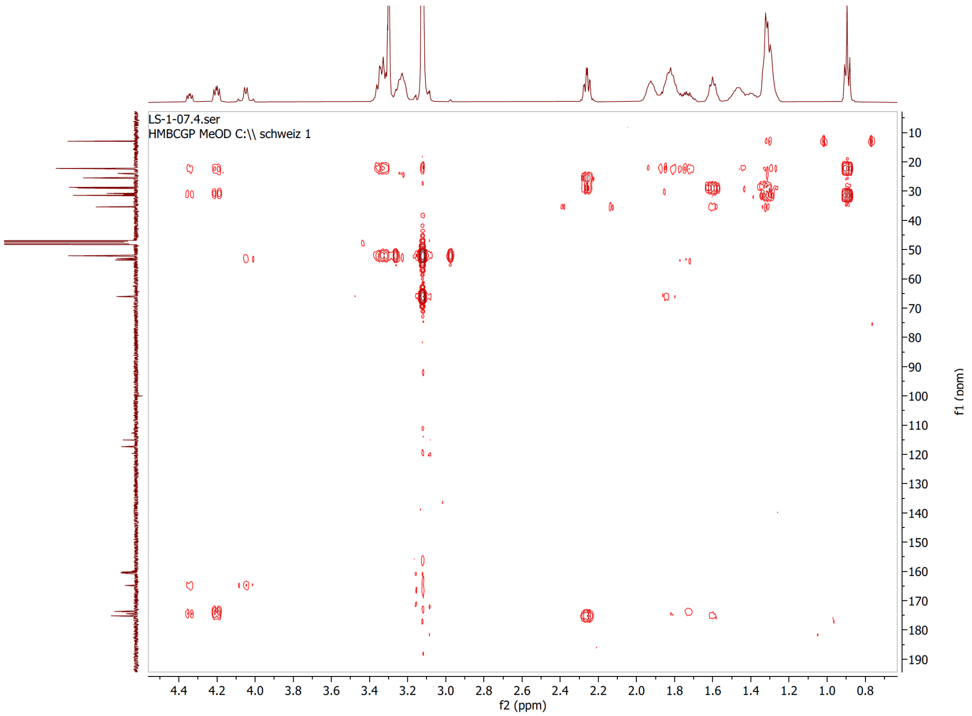

HMBC spectrum of UTBLP **4**.

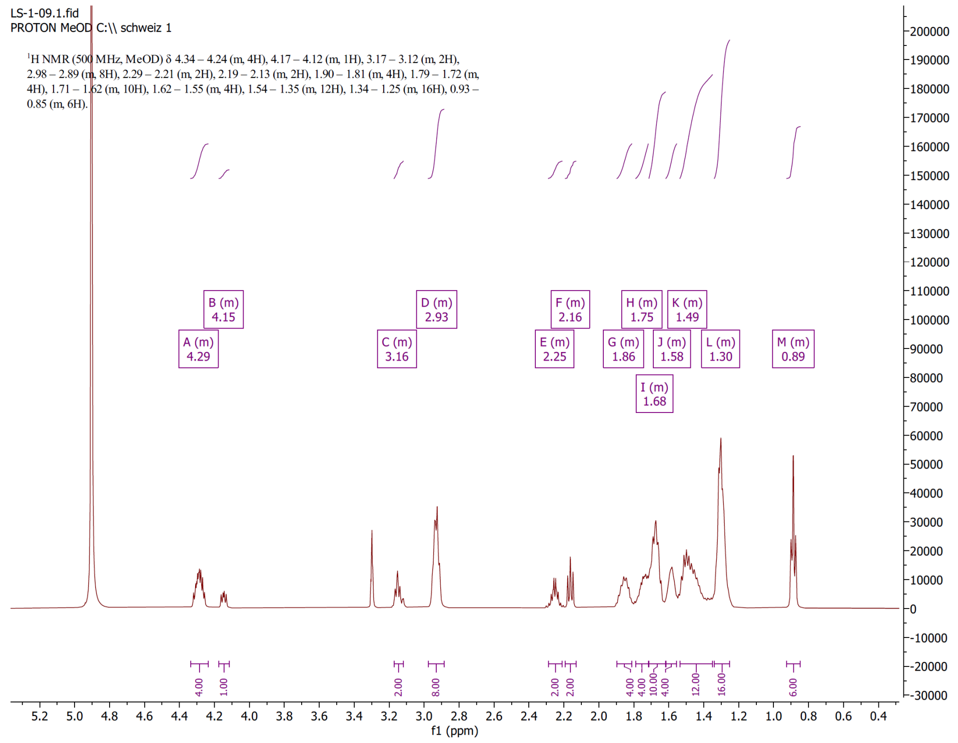

^{1}H spectrum of UTBLP **5**.

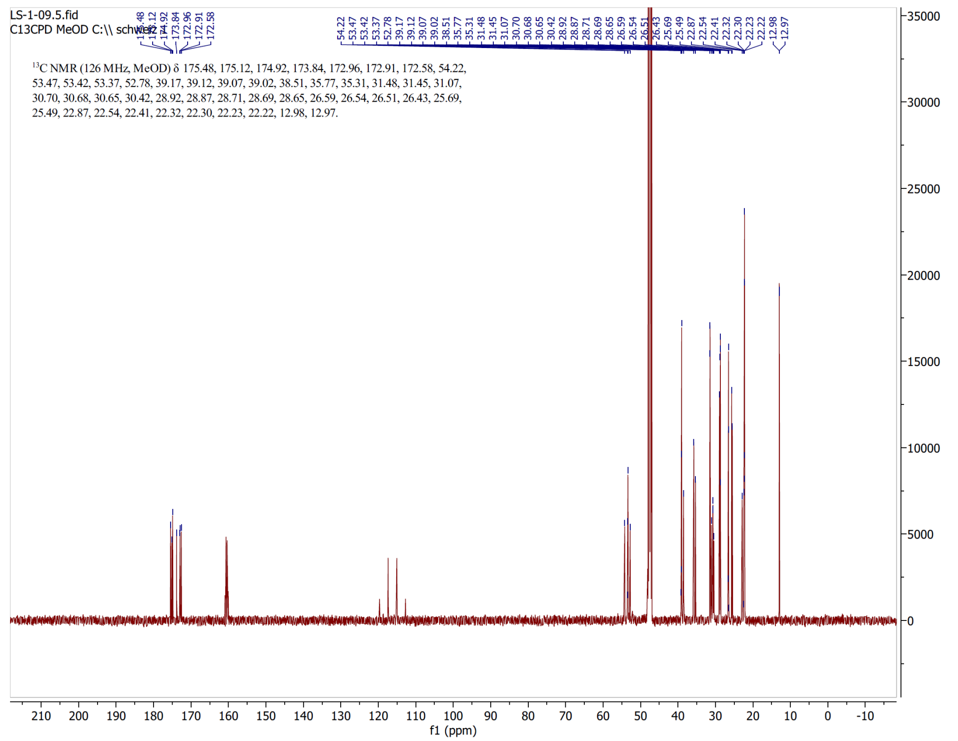

^{13}C spectrum of UTBLP **5**.

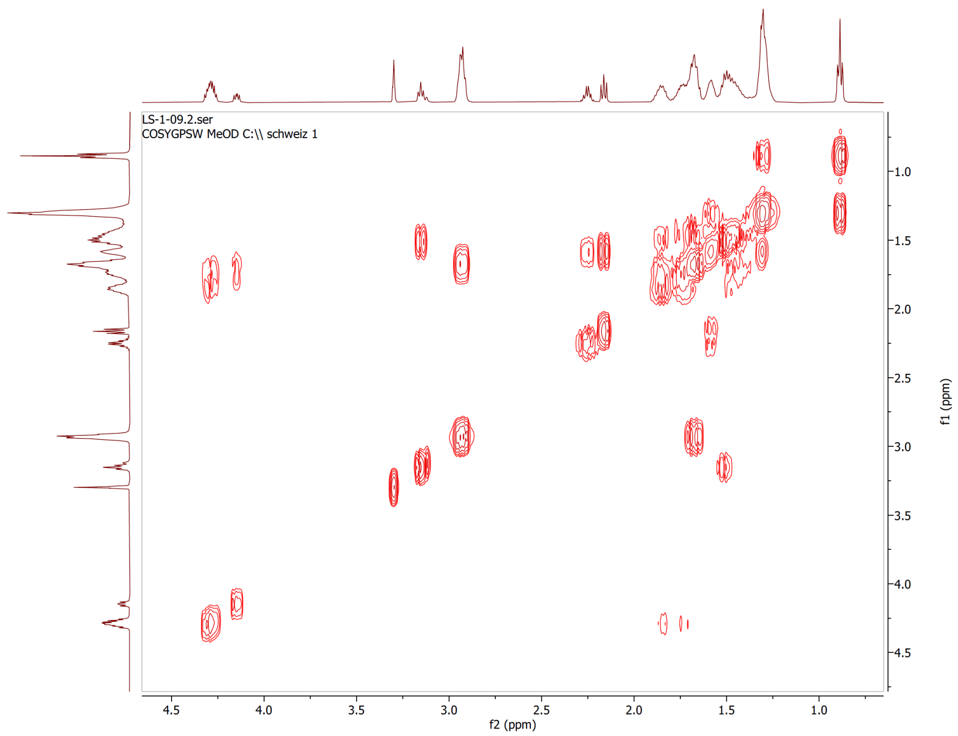

COSY spectrum of UTBLP **5**.

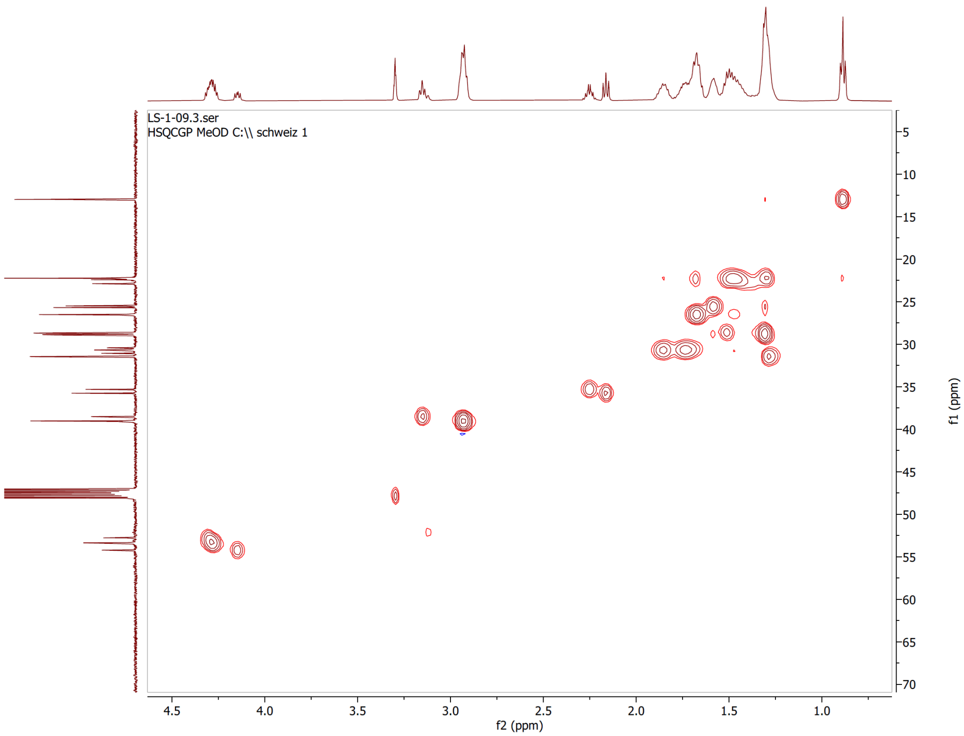

HSQC spectrum of UTBLP **5**.

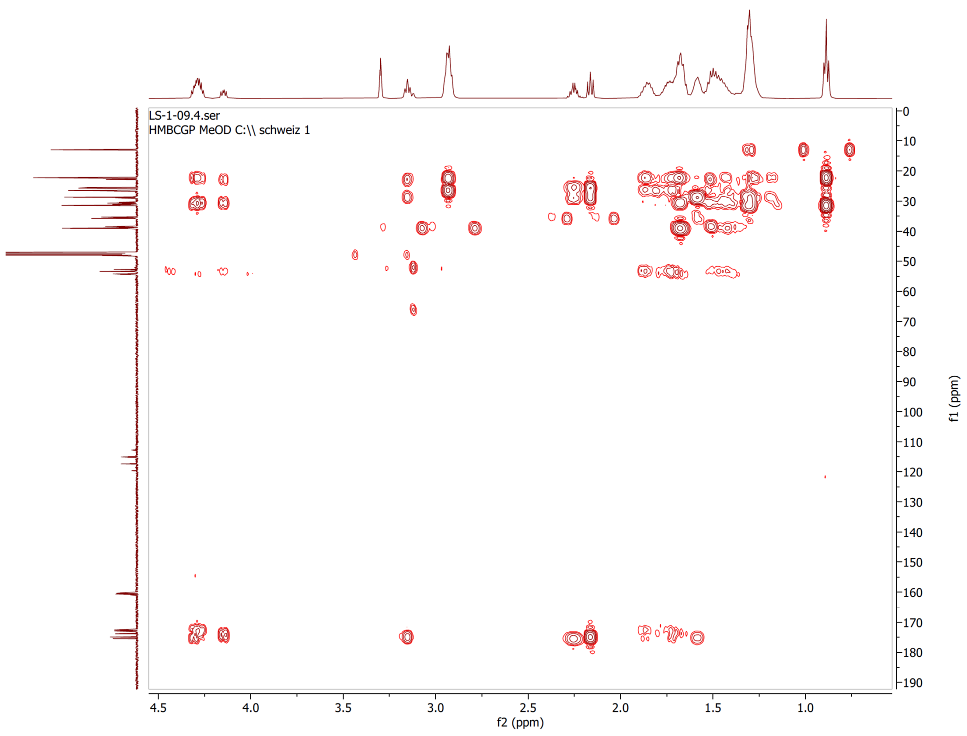

HMBC spectrum of UTBLP **5**.

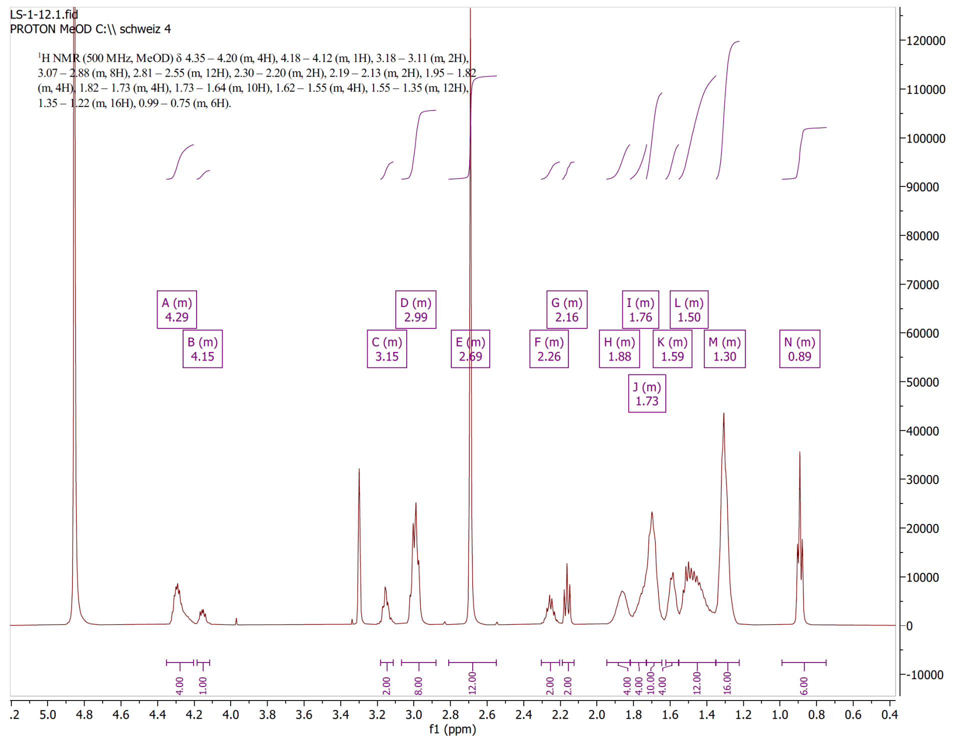

^{1}H spectrum of UTBLP **6**.

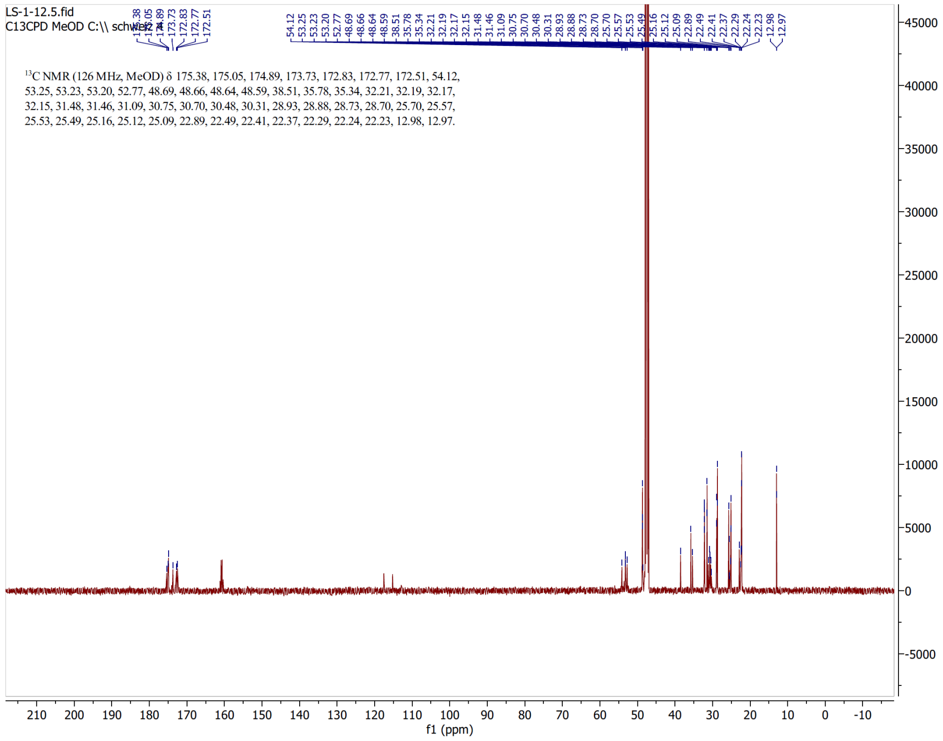

^{13}C spectrum of UTBLP **6**.

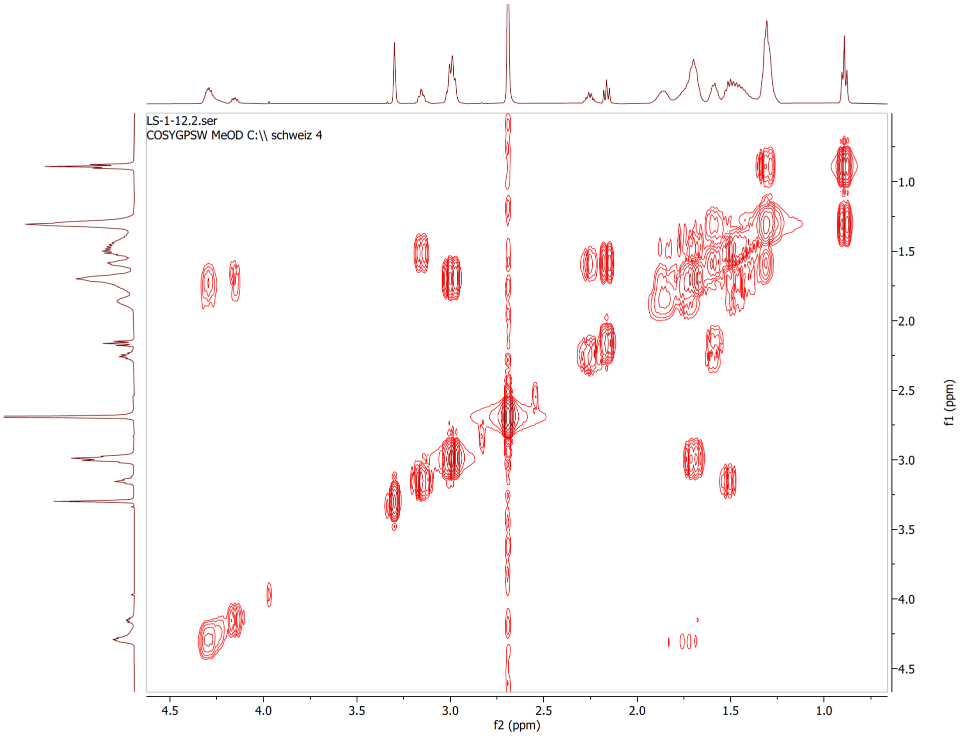

COSY spectrum of UTBLP **6**.

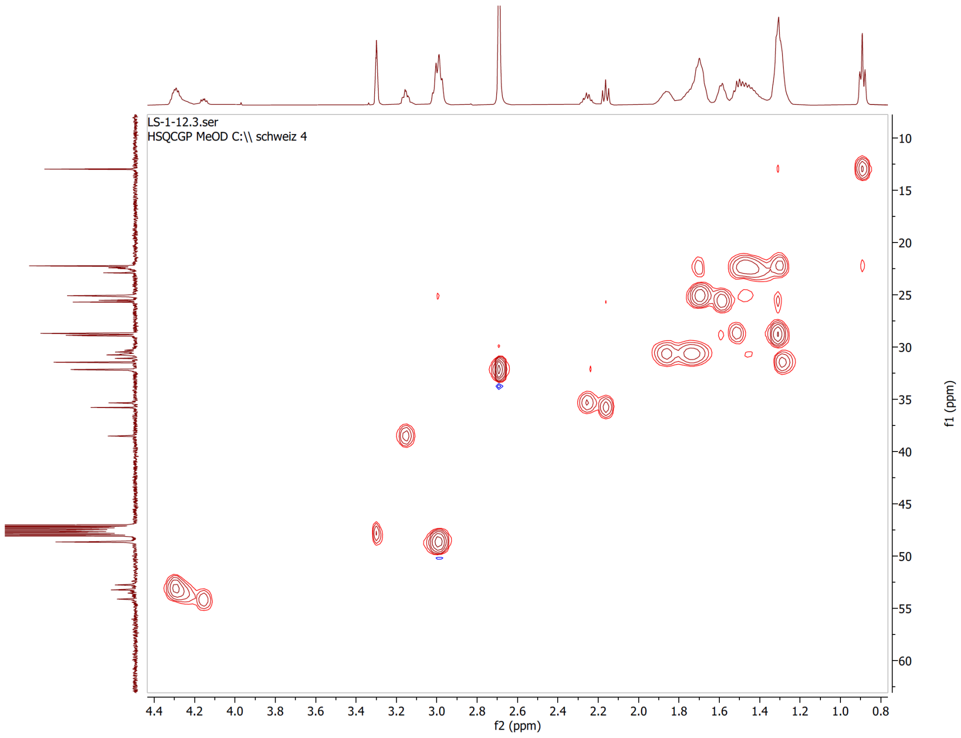

HSQC spectrum of UTBLP **6**.

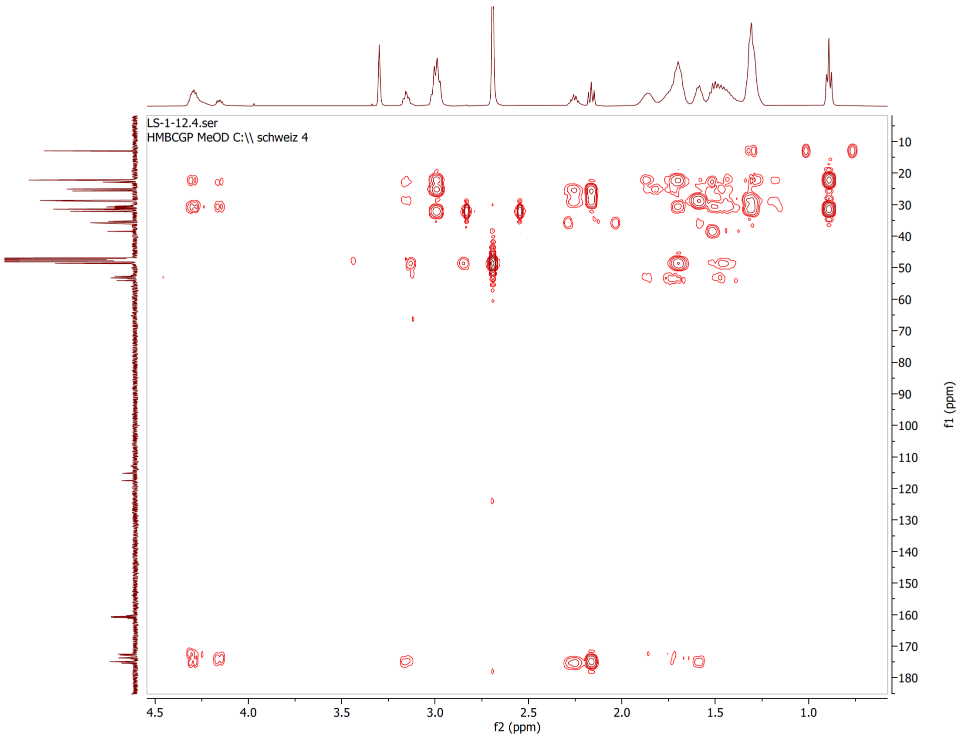

HMBC spectrum of UTBLP **6**.

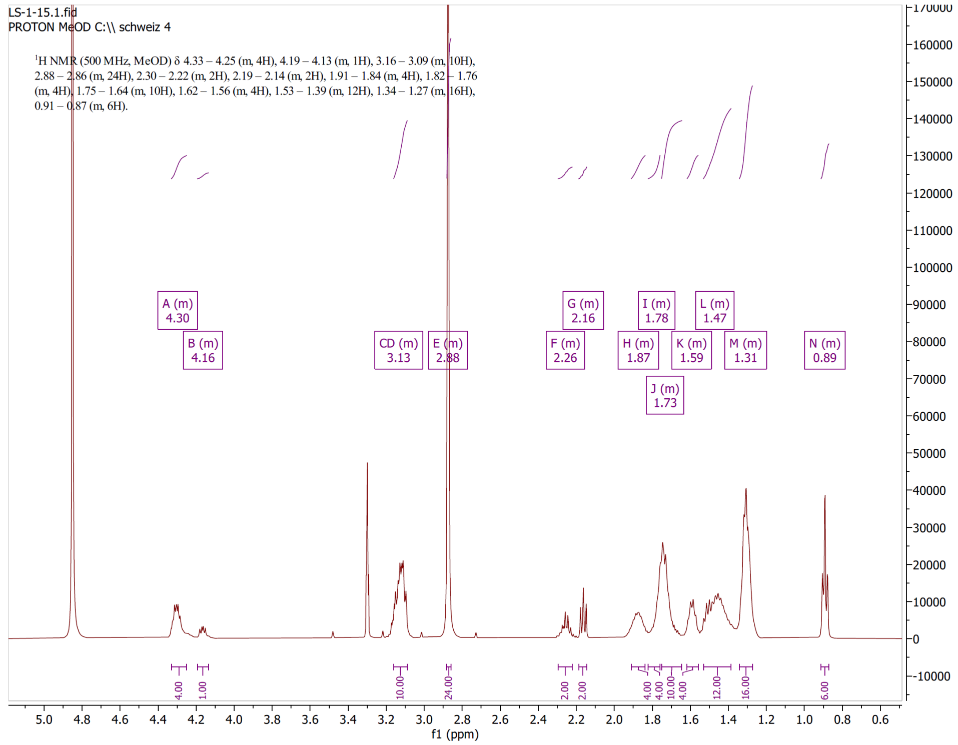

^{1}H spectrum of UTBLP **7**.

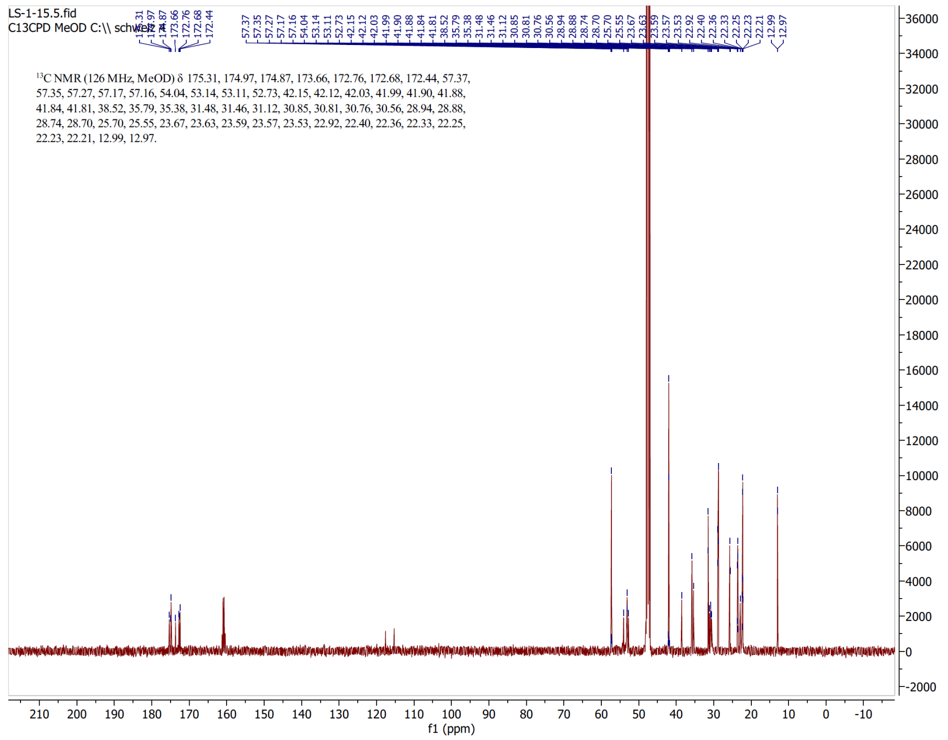

^{13}C spectrum of UTBLP **7**.

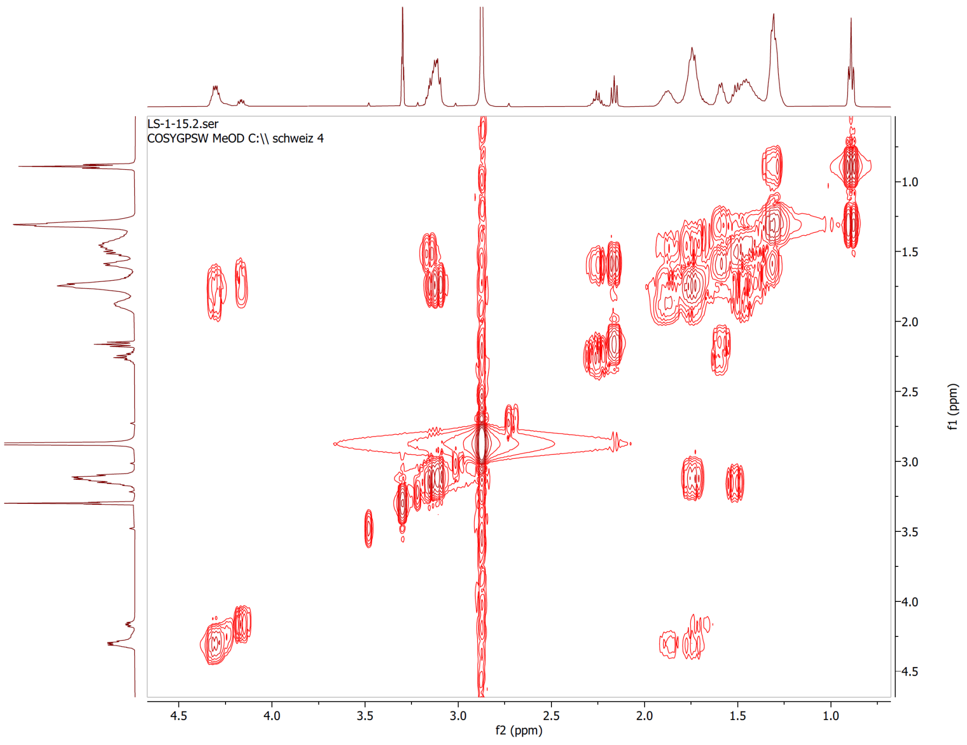

COSY spectrum of UTBLP **7**.

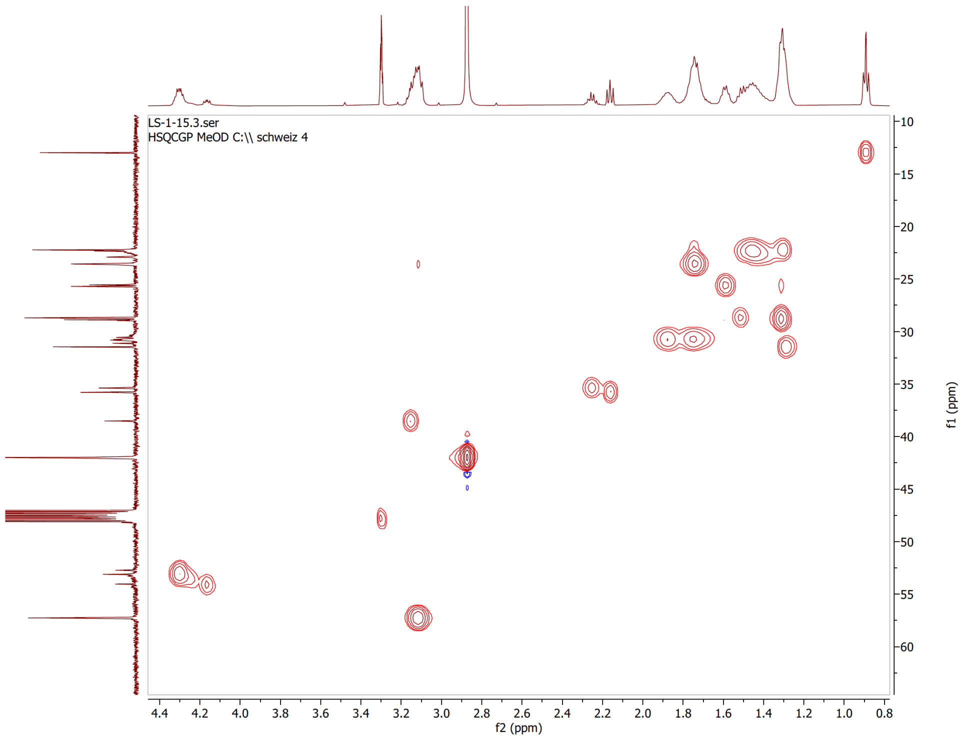

HSQC spectrum of UTBLP **7**.

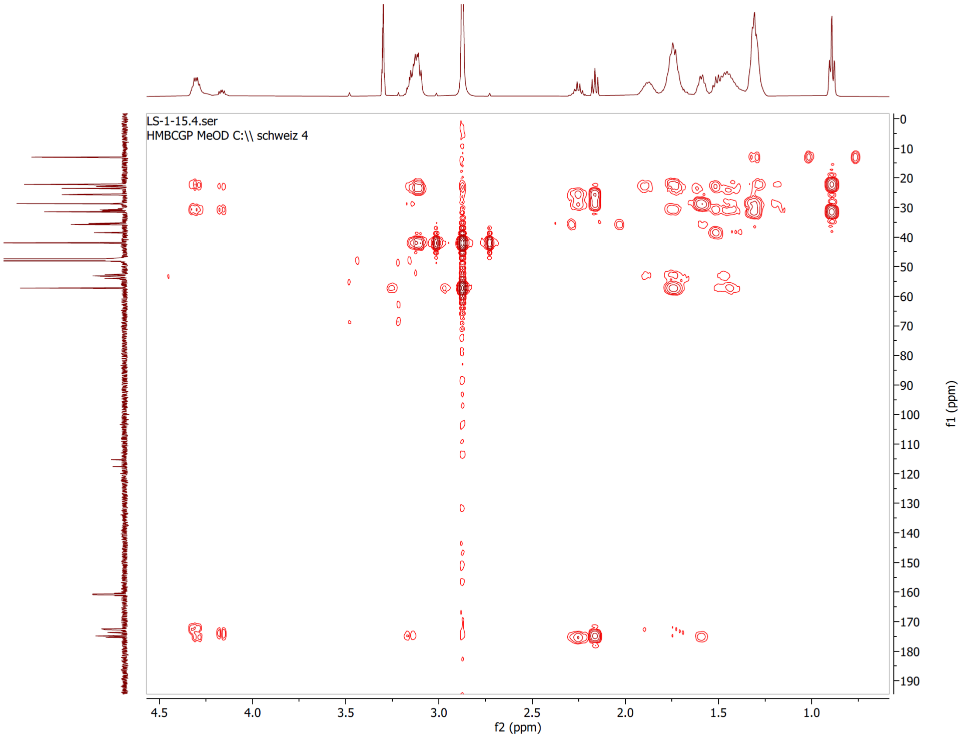

HMBC spectrum of UTBLP **7**.

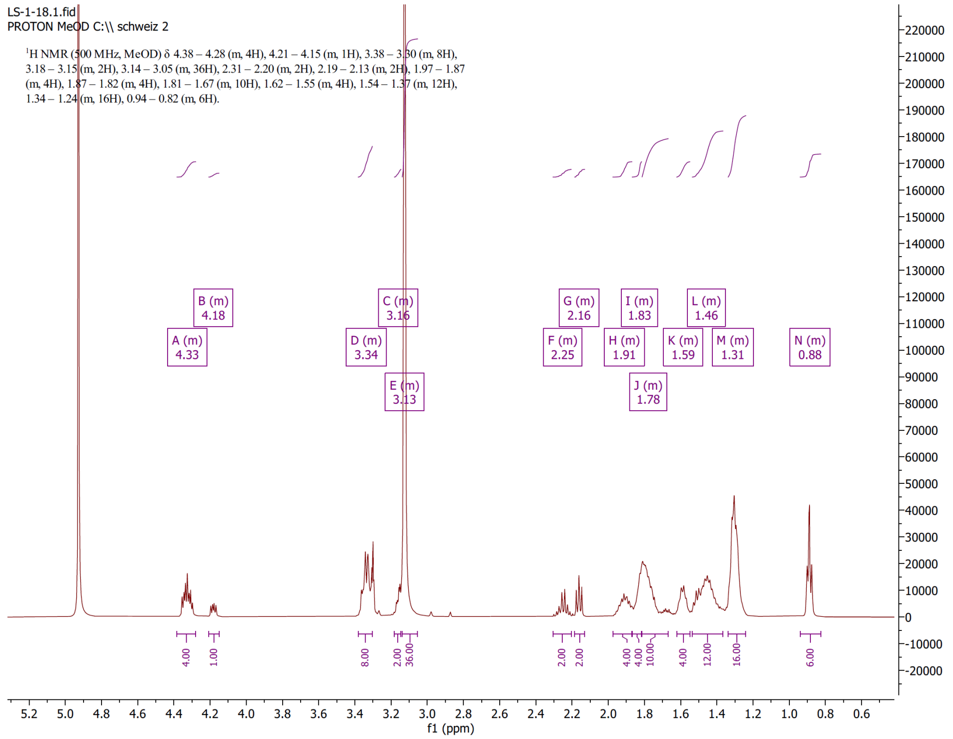

^{1}H spectrum of UTBLP **8**.

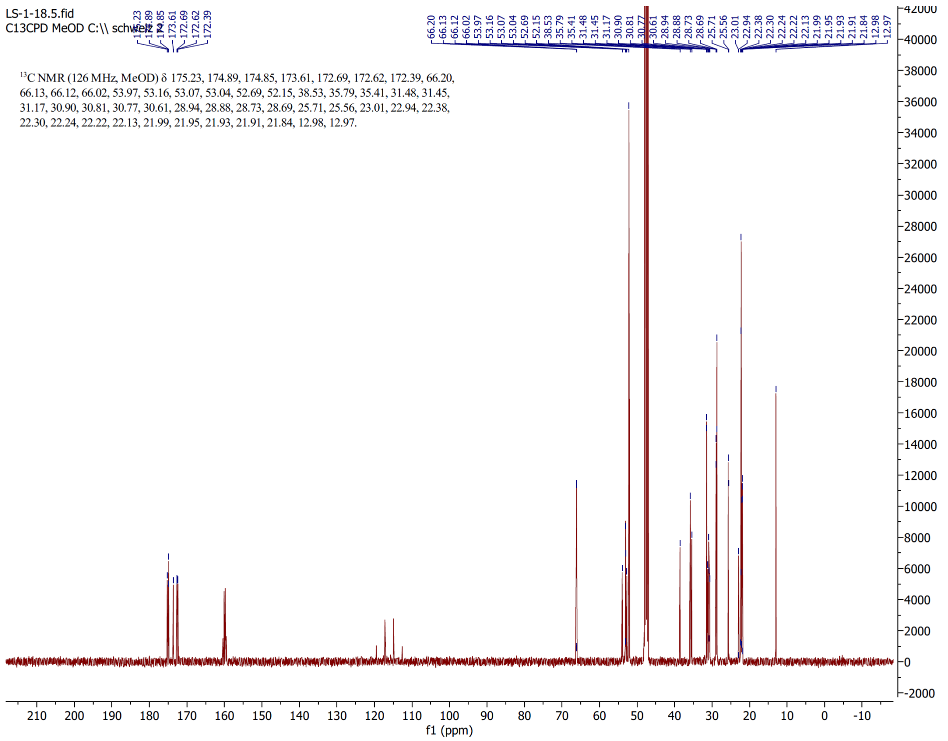

^{13}C spectrum of UTBLP **8**.

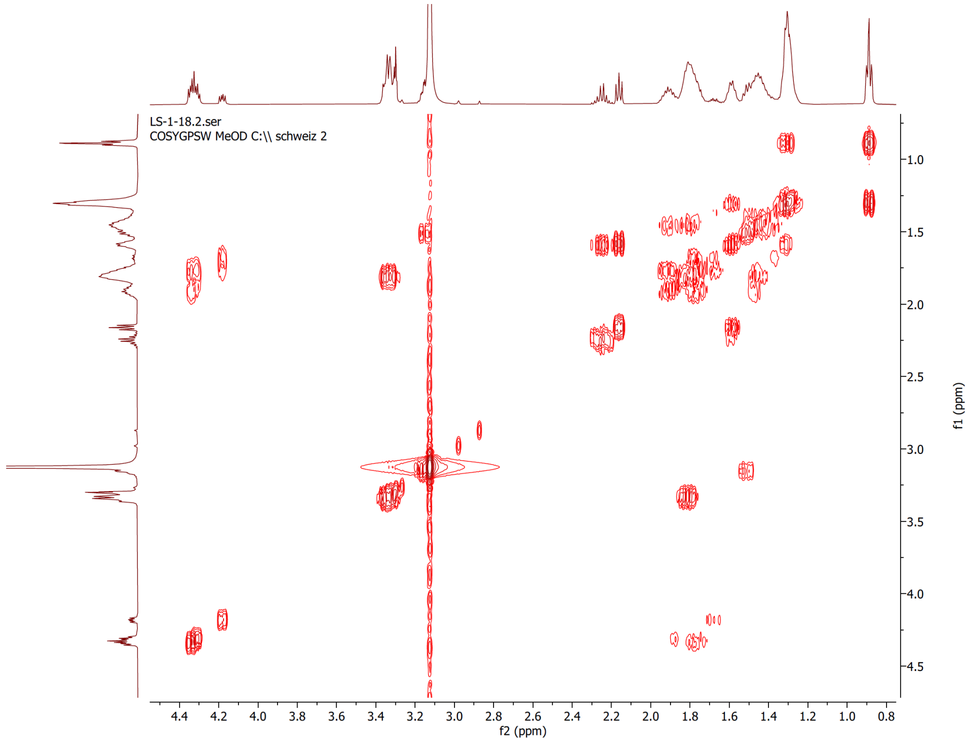

COSY spectrum of UTBLP **8**.

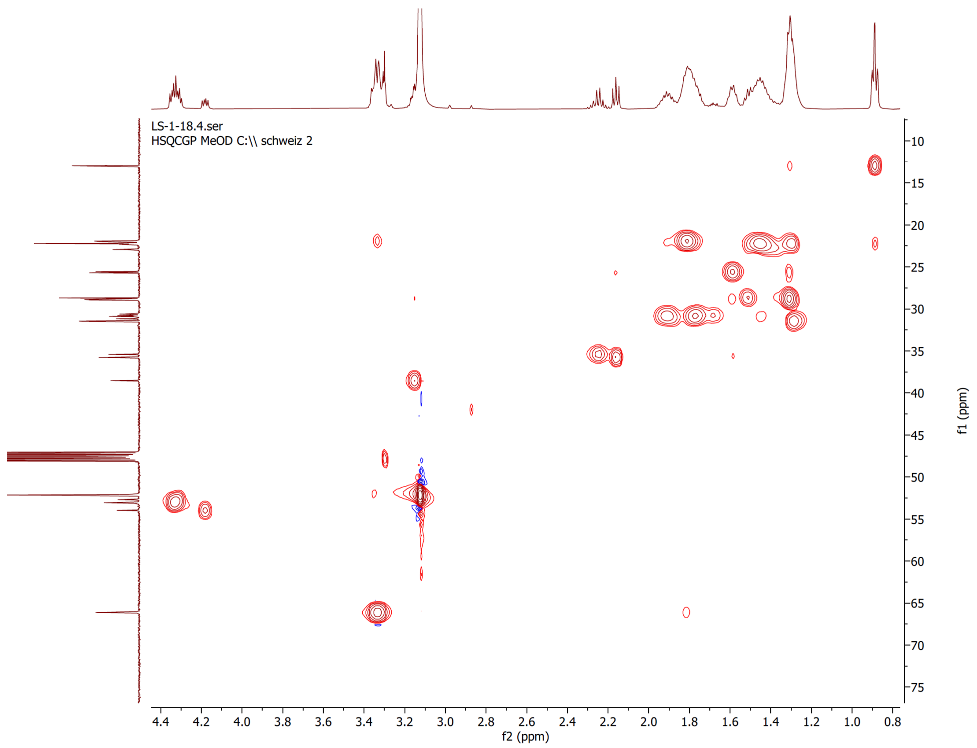

HSQC spectrum of UTBLP **8**.

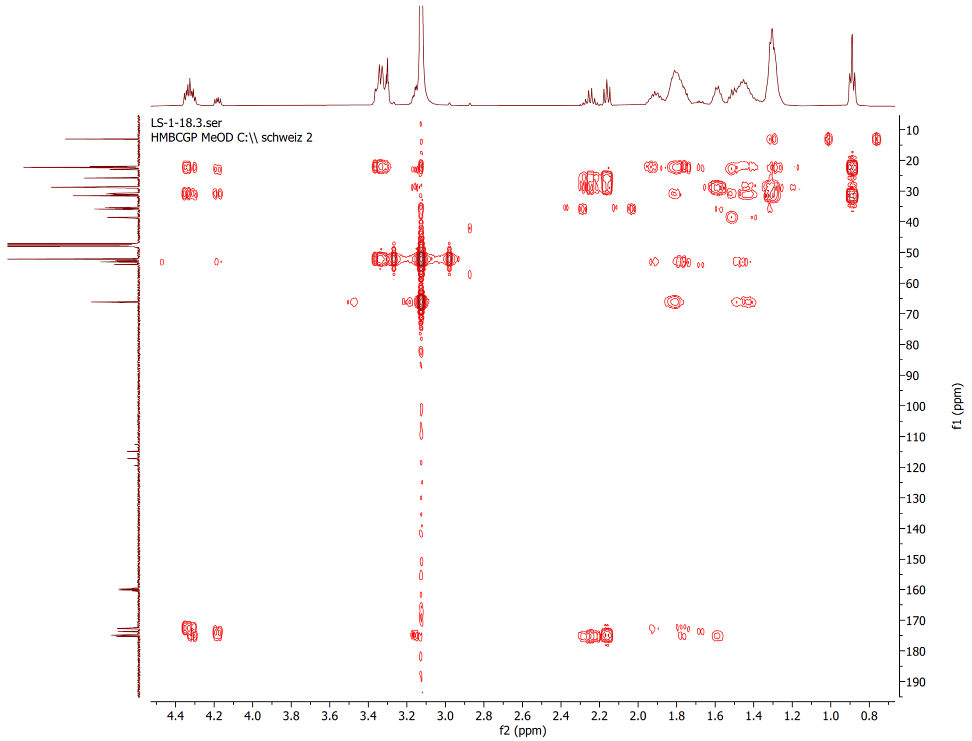

HMBC spectrum of UTBLP **8**.

Table S5. MICs (in μg/mL) of various antibiotics against MDR GNB used in the study.

P. aeruginosa	PTZ	A/C	AZT	FOX	CFZ	CTR	CPM	CTX	CAZ	IMI	MER	DOR	ETP	CIP	LEV	MOX	TOB	GEN	AMK	TGC	MIN	DOX	ERC	OMC	CST	CAM
259-96196	64	>32	32	>32	>128	>64	>64	2048	512	32	1024	>1024	>32	>16	256	>16	256	>32	>64	32	32	32	8	64	1	1024
262-101856	64	>32	32	>32	>128	64	32	128	16	32	32	16	>32	>16	64	>16	1024	>32	>64	32	64	1024	8	64	1	2048

A. baumannii	PTZ	FOX	CFZ	CPM	CTX	CAZ	C/T	IMI	MER	CIP	LEV	MOX	TOB	GEN	AMK	TGC	MIN	DOX	ERC	OMC	CST	CAM
AB027	512	ND	>128	>128	>256	ND	>16	32	16	>16	8	8	ND	32	>64	4	0.25	ND	0.5	1	0.25	128
92247	<1	32	128	4	ND	ND	2	ND	4	≤0.06	ND	ND	ND	ND	<1	0.25	0.125	ND	ND	ND	4	ND

E. coli	PTZ	A/C	AZT	FOX	CFZ	CPM	CAZ	C/T	IMI	MER	ETP	CIP	LEV	MOX	TOB	GEN	AMK	TGC	MIN	DOX	ERC	OMC	CST	CAM
94393 (mcr-1 +)	≤1	4	≤0.12	4	1	≤0.25	≤0.25	0.25	0.25	≤0.03	≤0.03	0.5	1	1	≤0.5	≤0.5	2	0.25	2	4	0.5	4	4	4
94474 (mcr-1 +)	16	>32	≤0.12	16	4	≤0.25	0.5	0.5	0.25	≤0.03	≤0.03	>16	32	16	32	16	2	1	64	>32	1	16	16	4

E. cloacae	PTZ	A/C	AZT	FOX	CFZ	CPM	CAZ	C/T	IMI	MER	ETP	CIP	MOX	TOB	GEN	AMK	TGC	MIN	DOX	ERC	OMC	CST	CAM
118564	2	>32	≤0.12	>32	>128	0.25	0.5	ND	ND	0.12	ND	0.06	0.12	1	1	2	ND	ND	4	ND	ND	>16	ND

K. pneumoniae	PTZ	A/C	AZT	FOX	CFZ	CPM	CAZ	C/T	IMI	MER	ETP	CIP	LEV	MOX	TOB	GEN	AMK	TGC	MIN	DOX	ERC	OMC	CST	CAM
113250	4	4	≤0.12	1	1	1	0.5	2	0.25	≤0.03	≤0.03	≤0.06	0.125	≤0.06	≤0.5	≤0.5	≤1	ND	2	2	1	2	>16	4

PTZ: piperacillin-tazobactam, A/C: amoxicillin-clavulanic acid, AZT: aztreonam, FOX: cefoxitin, CFZ: cefazolin, CTR: ceftriaxone, CPM: cefepime, CTX: cefotaxime, CAZ: ceftazidime, C/T: ceftolozane-tazobactam, IMI: imipenem, MER: meropenem, DOR: doripenem, ETP: ertapenem, CIP: ciprofloxacin, LEV: levofloxacin, MOX: moxifloxacin, TOB: tobramycin, GEN: gentamicin, AMK: amikacin, TGC: tigecycline, MIN: minocycline, DOX: doxycycline, ERC: eravacycline, OMC: omadacycline, CST: colistin, CAM: chloramphenicol, ND: not determined.
